# deCLUTTER^2+^ – a pipeline to analyze calcium traces in a stem cell model for ventral midbrain patterned astrocytes

**DOI:** 10.1242/dmm.049980

**Published:** 2023-06-23

**Authors:** Martyna M. Grochowska, Federico Ferraro, Ana Carreras Mascaro, Domenico Natale, Amber Winkelaar, Valerie Boumeester, Guido J. Breedveld, Vincenzo Bonifati, Wim Mandemakers

**Affiliations:** Erasmus MC, University Medical Center Rotterdam, Department of Clinical Genetics, P.O. Box 2040, 3000 CA Rotterdam, Netherlands

**Keywords:** Human astrocytes, Ventral midbrain, Induced pluripotent stem cells, Disease modeling, Parkinson's disease, Calcium imaging data analysis, Human stem cell-based models

## Abstract

Astrocytes are the most populous cell type of the human central nervous system and are essential for physiological brain function. Increasing evidence suggests multiple roles for astrocytes in Parkinson's disease, nudging a shift in the research focus, which historically pivoted around ventral midbrain dopaminergic neurons (vmDANs). Studying human astrocytes and other cell types *in vivo* remains challenging. However, *in vitro*-reprogrammed human stem cell-based models provide a promising alternative. Here, we describe a novel protocol for astrocyte differentiation from human stem cell-derived vmDAN-generating progenitors. This protocol simulates the regionalization, gliogenic switch, radial migration and final differentiation that occur in the developing human brain. We characterized the morphological, molecular and functional features of these ventral midbrain patterned astrocytes with a broad palette of techniques and identified novel candidate midbrain-astrocyte specific markers. In addition, we developed a new pipeline for calcium imaging data analysis called deCLUTTER^2+^ (deconvolution of Ca^2+^ fluorescent patterns) that can be used to discover spontaneous or cue-dependent patterns of Ca^2+^ transients. Altogether, our protocol enables the characterization of the functional properties of human ventral midbrain patterned astrocytes under physiological conditions and in disease.

## Teaser

**Summary:** We developed the deCLUTTER^2+^ pipeline to analyze calcium traces to discover spontaneous or cue-dependent patterns of Ca^2+^ transients, and a protocol for the generation of iPSC-derived ventral midbrain patterned astrocytes.

## INTRODUCTION

Astrocytes are specialized glial cells essential for the healthy functioning of the nervous system (Sofroniew and Vinters, 2010). Astrocytes release neurotrophic factors (Dezonne et al., 2013; Gomes et al., 1999, 2005; Nones et al., 2012), ensure the formation and maintenance of the blood-brain barrier (Verkhratsky and Nedergaard, 2018), regulate brain energy metabolism (Beard et al., 2021; González-Reyes et al., 2017), modulate synaptic activity (Chung et al., 2015), and allow the movement of fluid between the paravascular spaces and the interstitium (Jessen et al., 2015). In addition, astrocytes provide metabolic support to neurons, including the uptake and exchange of mitochondria (Scheibye-Knudsen et al., 2015) and lipids (Ioannou et al., 2019; Liu et al., 2015).

Accumulating evidence indicates that some of these homeostatic and neuronal health-promoting functions of astrocytes are impaired in Parkinson's disease (PD) (Booth et al., 2017; MacMahon Copas et al., 2021). The majority of PD research focuses on the vulnerable ventral midbrain dopaminergic neurons (vmDANs), the progressive loss of which is a disease hallmark. In recent years, however, the role of astrocytes in neurodegenerative processes has gained more attention. Studies have shown that genes known to cause inherited forms of PD are highly expressed in astrocytes and play vital roles in astrocyte function (Bandopadhyay et al., 2004; di Domenico et al., 2019; Grochowska et al., 2021; Kim et al., 2016, 2013; Qiao et al., 2016; Strokin et al., 2012). Moreover, in PD, astrocytes can acquire a neurotoxic phenotype, enhance neurodegeneration, and thus form a target for therapeutic intervention (Liddelow et al., 2017; Miyazaki and Asanuma, 2020; Schonhoff et al., 2020; Yun et al., 2018).

Astrocytes are a highly heterogeneous cell type based on their morphology, transcriptome and physiology (Batiuk et al., 2020; Bayraktar et al., 2020; Oberheim et al., 2009; Pestana et al., 2020; Siletti et al., 2022 preprint). They elicit heterogeneous responses to injury and functionally specialize to their surrounding tissue (Bugiani et al., 2022). Astrocytes residing in the ventral midbrain are physiologically distinct from, for example, astrocytes in the cortex and hippocampus (Siletti et al., 2022 preprint; Xin et al., 2019). Additionally, ventral midbrain astrocytes alleviate neuronal α-synuclein pathology, which is one of the main pathological hallmarks of PD (Yang et al., 2022).

Studying specific characteristics of human ventral midbrain astrocytes in PD remains challenging owing to limited access to primary human cells from the affected brain regions. However, using induced pluripotent stem cell (iPSC) technology, it is now possible to generate various astrocyte populations of human origin *in vitro* (Lanjewar and Sloan, 2021). Evidence supports that patterning of iPSC-derived progenitors to rostral-caudal and dorsal-ventral identities with the same morphogens used for the neuronal subtype specification generates region-specific astroglial subtypes (Krencik et al., 2011).

A limited number of protocols exist that allow the generation of ventral midbrain patterned astrocytes (Table S1). A feature shared by these protocols is the generation of progenitor cells patterned toward a mesencephalic fate (ventral neural tube progenitors) that can also differentiate into vmDANs. Although these models have been an advancement in the PD research field, modifications and improved characterization remain necessary to generate more accurate models of human ventral midbrain astrocytes.

Here, we describe a novel protocol for generating ventral midbrain patterned astrocytes from iPSCs. Similar to previous studies, we rely on the ventral neural tube patterning of iPSC-derived neuroepithelial cells to generate ventral midbrain patterned astrocytes from a population of progenitors that can also produce vmDANs (Reinhardt et al., 2013). Our protocol mimics gliogenesis *in vitro*, including regionalization, gliogenic switch, radial migration and final differentiation. In comparison to previous studies, our simplified protocol efficiently generates mature ventral midbrain astrocytes relatively fast, without the use of serum or transgenic reporters, and a limited number of necessary additives. We characterized these astrocytes using immunocytochemistry (ICC), RNA-sequencing (RNA-seq) and a repertoire of functional assays, including pro-inflammatory cytokine treatment, glutamate uptake and fluorescence time-lapse calcium confocal imaging. Furthermore, we developed a novel pipeline for the functional analysis of calcium imaging data from *in vitro* experiments that we named deCLUTTER^2+^ (deconvolution of Ca^2+^ fluorescent patterns). Altogether, our model can potentially study the properties of ventral midbrain patterned astrocytes under physiological conditions and in disease.

## RESULTS

### Astrocytes are derived from ventral midbrain patterned progenitor cells

We developed a strategy to differentiate human-derived iPSCs into astrocytes by combining various steps from previously described astrocyte differentiation protocols and the principles of astrocyte development *in vivo*. First, we generated eight iPSC lines that formed compact colonies with well-defined edges and expressed pluripotency markers, including the proteins NANOG and OCT-4 (encoded by *POU5F1*), the glycosphingolipid SSEA-4, and the epitope recognized by antibody TRA-1-81 (Fig. S1A). Based on the ICC data, there were no apparent differences in the expression of these pluripotency markers among the lines. In addition, we tested the expression of pluripotency markers with real-time quantitative PCR (RT-qPCR) (Fig. S1B-E). We detected the expression of pluripotency markers in all generated iPSC lines. However, the lines differed in the expression of pluripotency markers from the reference line HUES9. Next, we generated neural progenitor cells patterned towards a ventral neural tube identity that are able to also differentiate into dopaminergic neurons (Fig. S1F-H) as previously described (Grochowska et al., 2021; Monzel et al., 2017; Quadri et al., 2018; Reinhardt et al., 2013; Vanhauwaert et al., 2017). These progenitor lines expressed neural progenitor markers such as SOX2 and nestin (NES) but remained undifferentiated, as demonstrated by low expression of the mature neuron marker MAP2 (Fig. S1H). Moreover, we assessed the expression of gene sets that are crucial during mammalian brain development (de Rus Jacquet, 2019; Kirkeby et al., 2012; Nolbrant et al., 2017). The transcriptomic analysis of progenitor cells showed high expression of markers specific for the caudal fate (midbrain and hindbrain), including *GBX2*, *HOXA2* and *IRX3*. In addition, the low expression of rostral markers (diencephalon and telencephalon) confirmed the caudal identity of these progenitor cells (Fig. S2). We also demonstrate elevated expression of the floor plate-specific markers (*FOXA2* and *NTN1*) and midbrain-specific markers (*EN1*, *EN2*, *PAX5* and *PAX6*) in several progenitor lines cells (Fig. S2).

**Fig. 1.**
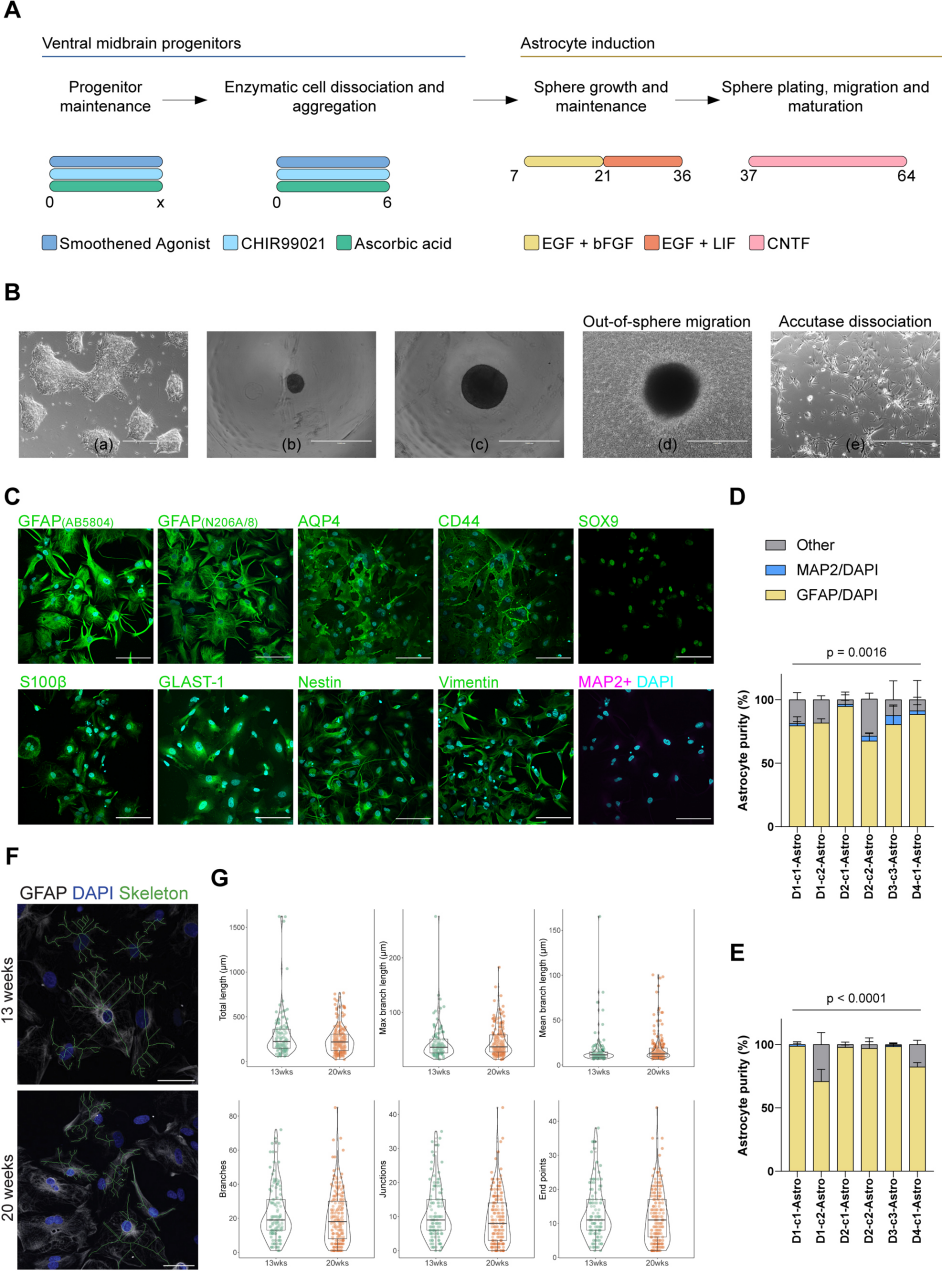
**Generation and immunocytochemical characterization of astrocytes derived from ventral midbrain patterned progenitors.** (A) Schematic of the astrocyte differentiation protocol depicting the major steps with the accompanying medium supplements. Numbers indicate days. (B) Representative bright-field images of various cell morphologies at different culturing steps during differentiation. Ventral midbrain progenitors (a) were seeded in low attachment plates to form spheres (b). Spheres were cultured for several weeks in EGF/bFGF- or EGF/LIF-containing media (c). Spheres were plated and further differentiated with CNTF (d). Once matured, astrocytes were enzymatically dissociated and used for downstream assays (e). Scale bars: 400 μm. (C) Representative ICC images of 20-week-old astrocytes staining positive for the general astrocyte markers GFAP, AQP4, SOX9, S100β and GLAST-1, and for the astrocytic precursor markers CD44, vimentin, and nestin. Astrocytes were negative for the mature neuron marker MAP2. Nuclei were counterstained with DAPI (cyan). Scale bars: 100 μm. (D,E) Histograms show the percentage of MAP2- or GFAP-positive cells to the total number of cells (DAPI). Astrocyte cultures mainly comprised GFAP-positive cells at 13 weeks (D) and 20 weeks (E). Three independent differentiations for lines D1-c1, D1-c2, D2-c1 and D2-c2 were analyzed, and two independent differentiations for lines D3-c3 and D4-c1 were analyzed. Two-way ANOVA was used to determine the interaction *P*-value. Data show the mean±s.d. (F) Representative ICC images of 13- and 20-week-old astrocytes staining positive for the general astrocyte marker GFAP (gray), with reconstructed skeletons for morphological characterization (green). Nuclei were counterstained with DAPI (cyan). Scale bars: 50 μm. (G) Violin and box plots showing the distributions of morphological characteristics of 13- and 20-week-old astrocytes. Total length, max branch length and mean branch length are shown in micrometers; the numbers of branches, junctions and end points are reported as counts. Boxes represent the 25–75th percentiles, whiskers show the interquartile range×1.5, and the median is marked with a line. Images are representative of one differentiation of the six lines.

*In vivo*, neural progenitors undergo many rounds of neurogenesis before committing to the glial fate (Miller and Gauthier, 2007). Hence, we strived to accelerate gliogenesis *in vitro* by combining several well-established approaches (Barbar et al., 2020; Boissart et al., 2012, 2013; Krencik and Zhang, 2011; Lattke et al., 2021; Monzel et al., 2017; Perriot et al., 2018; Reinhardt et al., 2013). It has been shown that culturing astrocytes in a three-dimensional matrix induces the expression of astrocyte-specific genes and improves astrocyte maturity (Lattke et al., 2021). Therefore, we aggregated progenitor cells to form floating spheres (Fig. 1A,Ba,Bb,Bc). Next, we exposed the spheres to basic fibroblast growth factor (bFGF, encoded by *FGF2*) and epidermal growth factor (EGF) to mass amplify progenitor cells. Consecutively, we added leukemia inhibitory factor (LIF) and EGF to accelerate glial differentiation by the activation of the JAK-STAT signaling pathway (Bonni et al., 1997; Perriot et al., 2018) (Fig. 1A). Finally, when astrocyte-enriched spheres were plated, astrocytes migrated radially out of the spheres in the presence of ciliary neurotrophic factor (CNTF) for further maturation (Fig. 1A,Bd). Out of eight cell lines, two lines failed to attach firmly to the plates. Therefore, they were excluded from further analyses. This procedure led to a proliferative population of astrocytes expressing key astrocyte markers, including glial fibrillary acidic protein (GFAP), aquaporin 4 (AQP4), SRY-box transcription factor 9 (SOX9), S100 calcium-binding protein β (S100β, encoded by *S100B*), glutamate transporter (GLAST-1, encoded by *SLC1A3*), and the astrocytic precursor markers CD44, nestin and vimentin (VIM) in all human iPSC-derived astrocytes as demonstrated by ICC (Fig. 1C; Fig. S3). On average, this protocol yielded 82% of GFAP-expressing astrocytes at 13 weeks in culture (Fig. 1D). The proportion of GFAP-expressing astrocytes increased to 90% after 20 weeks in culture (Fig. 1E). At both time points, there were significant differences detected in cell composition between lines (Fig. 1D,E). No evident contamination by neurons was found, as assessed by immunostaining with an anti-MAP2 antibody, confirming the efficiency of this protocol in generating highly enriched astrocyte populations (Fig. 1C-E).

In addition, we morphologically characterized the astrocytes stained with GFAP using the ImageJ plugin ‘Skeletonize 2D/3D’ at 13 and 20 weeks (Fig. 1F,G). At 13 weeks, we characterized a total of 122 astrocytes, including at least ten for each of the six astrocyte lines. At 20 weeks, we characterized 189 astrocytes from the same six lines previously analyzed, with at least 12 astrocytes per line. No statistically significant difference was found when comparing the assessed metrics across the two time points.

### Genomic integrity analyses of iPSC-derived cells

Prolonged culturing exposes cells to strong selection pressures, often resulting in genomic alterations. Further manipulation of these cells may also jeopardize their genomic stability (Weissbein et al., 2016). Therefore, before investigating the molecular characteristics of the generated cell lines, we performed additional quality-control steps.

First, as genomic DNA from the astrocyte lines used for these experiments was not available, we employed an alternative method to check for chromosomal abnormalities. This method is called eSNP-karyotyping and can be performed with the available RNA-seq data when traditional approaches cannot be used owing to the lack of material (Weissbein et al., 2016). We sequenced RNA from eight progenitor and six astrocyte lines. eSNP-karyotyping revealed a chromosome 2 multiplication in the astrocyte line D4-c1, even when the respective progenitor line appeared normal (Fig. S4).

Next, considering that eSNP-karyotyping is not a standard procedure for determining genomic integrity, we wanted to rule out any karyotype irregularities that might have been missed using this technique. We performed high-resolution array karyotyping on the available genomic DNA of the progenitors (Fig. S5). We confirmed the chromosome 2 multiplication found by eSNP-karyotyping in the astrocyte line D4-c1 and identified additional aberrations in other chromosomes (i.e. chromosomes 4, 8 and 17) in this cell line. Moreover, the high-resolution array karyotyping also pinpointed aberrations in the D1-c1 line (i.e. chromosomes 6 and 17) that were undetected by eSNP-karyotyping. Hence, we excluded the D1-c1 and D4-c1 lines from the following analyses.

### RNA expression profiling of ventral midbrain patterned progenitor cells and astrocytes

**Fig. 2.**
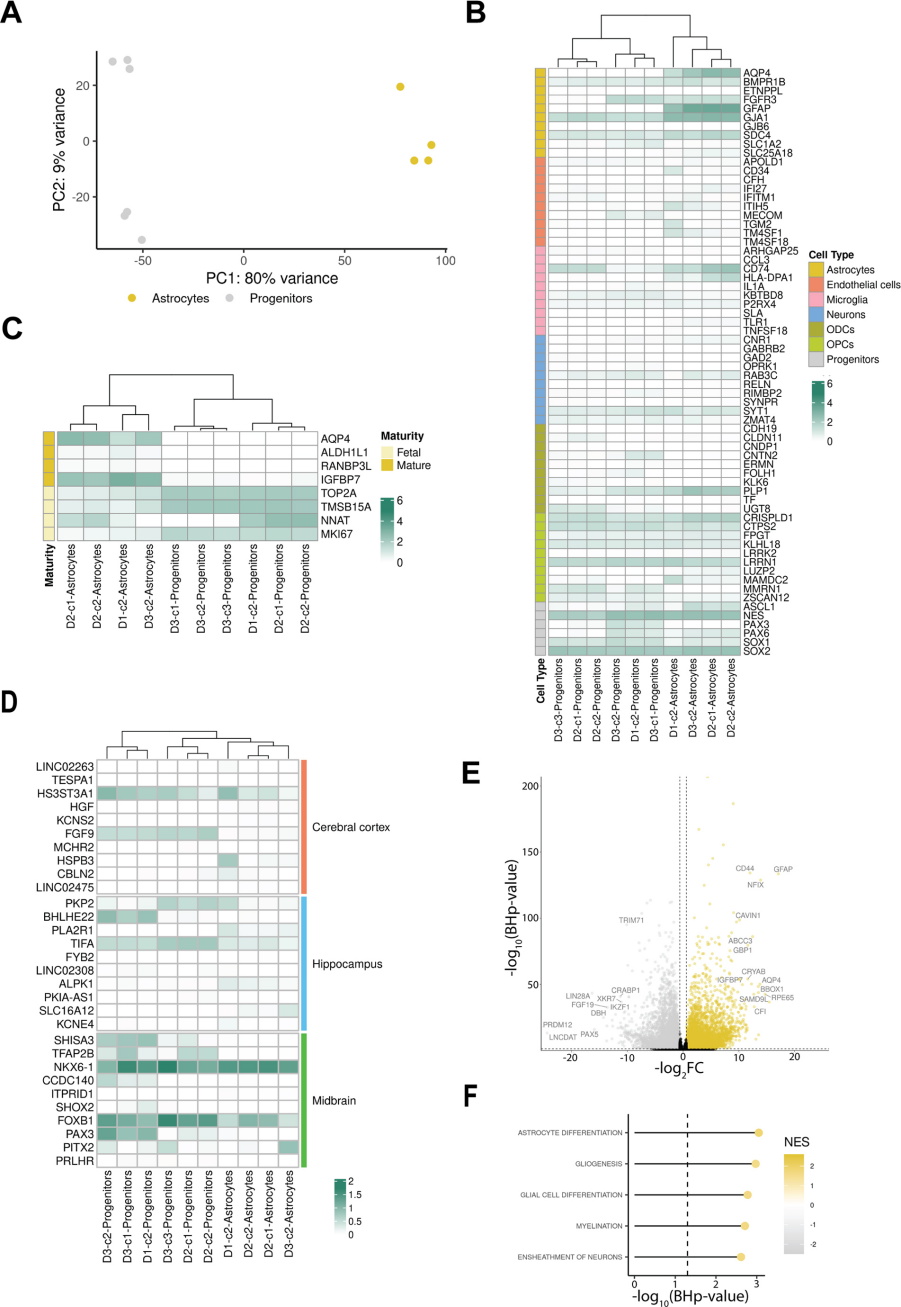
**Transcriptomic characterization of progenitors and astrocytes.** (A) PCA plot showing line clustering conducted on the top 500 variable genes. (B) Heatmap showing the level of expression as log_10_[fragments per kilobase of transcript per million mapped fragments (FPKM)+1] of the top ten BRETIGEA- and literature-described markers for brain cell types. ODC, oligodendrocytes; OPC, oligodendrocyte precursor cells. (C) Heatmap showing the level of expression as log_10_(FPKM+1) of literature-derived fetal and mature astrocyte markers. (D) Heatmap showing the level of expression as log_10_(FPKM+1) of the top ten candidate marker genes for the cerebral cortex, hippocampal and midbrain astrocytes. (E) Volcano plot showing significantly upregulated (yellow) and downregulated (gray) genes in the astrocyte lines compared to the progenitor lines. (F) Lollipop plot showing the top five (ordered by *BHp-*value) significantly enriched Gene Ontology biological processes among the ‘nervous system development’ (GO:0007399) child terms. Data are from one differentiation. NES, normalized enrichment score.

We employed our RNA-seq data to determine whether expression profiling could stratify the generated cell lines in an unbiased manner. Principal component analysis (PCA) on the top 500 most variable genes revealed two distinct groups corresponding to the two populations. Principal component 1 (PC1), capturing 80% of the variability in gene expression, clearly discriminated between the two populations (Fig. 2A).

Next, we assessed the expression of the top ten markers selected from the R package BRETIGEA (McKenzie et al., 2018; https://github.com/andymckenzie/BRETIGEA) encompassing six cell types (astrocytes, endothelial cells, neurons, microglia, oligodendrocytes and oligodendrocyte precursor cells) and from the literature for the progenitors to identify the cell lines (Sloan et al., 2017). The precursors and the astrocytes expressed the expected cell type-specific markers consistently. In contrast, lower to no expression of the majority of markers of the other cell types could be detected (Fig. 2B). In addition, we assessed the expression of genes known to play vital functions in astrocyte biology (Lovatt et al., 2007; Sloan et al., 2017). We found elevated expression of genes that play important roles in the regulation of the blood-brain barrier (*AQP4*, *EDNRB* and *MLC1*), neurotransmitter recycling (*SLC1A3* and *SLC4A4*), metabolic processes (*ALDH1L1* and *S100B*) and synaptogenesis (*SPARCL1*) in astrocytes but not in progenitor cells (Fig. S6). These findings were consistent across six different lines.

The astrocytes expressed some of the markers of the precursor cells. For this reason, we assessed the maturity level of the generated astrocytes using a list of markers for fetal and mature astrocytes (Sloan et al., 2017) (Fig. 2C). Compared to the precursor cells, astrocytes expressed a high level of two of the mature astrocyte markers (*AQP4* and *IGFBP7*). Lower expression levels of fetal astrocyte markers could still be detected in the astrocytes.

In order to evaluate the successful regionalization of our astrocytes, we determined the expression levels of candidate markers of region-specific astrocytes. We considered astrocytes from the adult human midbrain, cerebral cortex and hippocampus as they are physiologically distinct (Xin et al., 2019). We obtained these candidate markers by re-analyzing recently published single-nucleus RNA-seq (snRNA-seq) data (Siletti et al., 2022 preprint). The pseudo-bulk region-specific samples appeared clearly separated along PC1, with the clustering seemingly driven by the brain region rather than donor effects (Fig. S7). Differential expression analysis for each of these region-specific datasets against the others and the selection of the top significantly upregulated genes revealed candidate region-specific markers. The astrocytes produced by our protocol expressed higher levels of some midbrain astrocyte candidate markers (*NKX6-1* and *FOXB1*) but lower levels of some candidate markers of the hippocampus or cerebral cortex (Fig. 2D).

### Genome-wide expression profiling confirms effective differentiation

We performed differential expression analysis on the RNA-seq data to identify the genes driving the differences between the precursors and astrocytes we generated. A total of 12,154 differentially expressed genes (DEGs) were detected. Out of these, 6507 were upregulated in the astrocytes compared to in the progenitors (Fig. 2E; Table S6). Among the top upregulated genes, we found established astrocytic markers, including *GFAP* [log_2_(fold change or FC)±s.e.m. of log_2_FC=17.10±0.68, Benjamini–Hochberg-corrected *P-*value (*BHp*-value)=2.76×10^−134^] and *AQP4* (log_2_FC=13.79±0.90, *BHp*-value=7.39×10^−51^). Similarly, among the top downregulated genes, we identified genes related to the stemness and proliferative potential of the precursor cells, including *LIN28A* (log_2_FC=−16.33±1.15, *BHp*-value=5.09×10^−44^) and *PAX5* (log_2_FC=−15.91±1.85, *BHp*-value=1.18×10^−16^).

We also assessed the effect of the differentiation at the pathway level by performing gene set enrichment analysis (GSEA) on the genes ranked by the differential expression analysis statistics. We found that 1431 terms from the Gene Ontology biological process category were significantly different between the two cell types, with 945 being upregulated in the astrocytes, confirming broad differences in the transcriptomes of the two lines (Table S7). We prioritized the ‘nervous system development’ (GO:0007399) child terms from this category to focus on the differentiation effect. Of the 182 terms passing our filtering criteria, 22 significantly differed between astrocytes and precursors. The top significantly upregulated term was ‘astrocyte differentiation’ (normalized enrichment score=1.83, *BHp*-value=8.91×10^−4^), further supporting the directionality and effectiveness of the differentiation protocol (Fig. 2F; Table S7).

### Astrocytes respond to pro-inflammatory cytokines and display low extracellular glutamate uptake

**Fig. 3.**
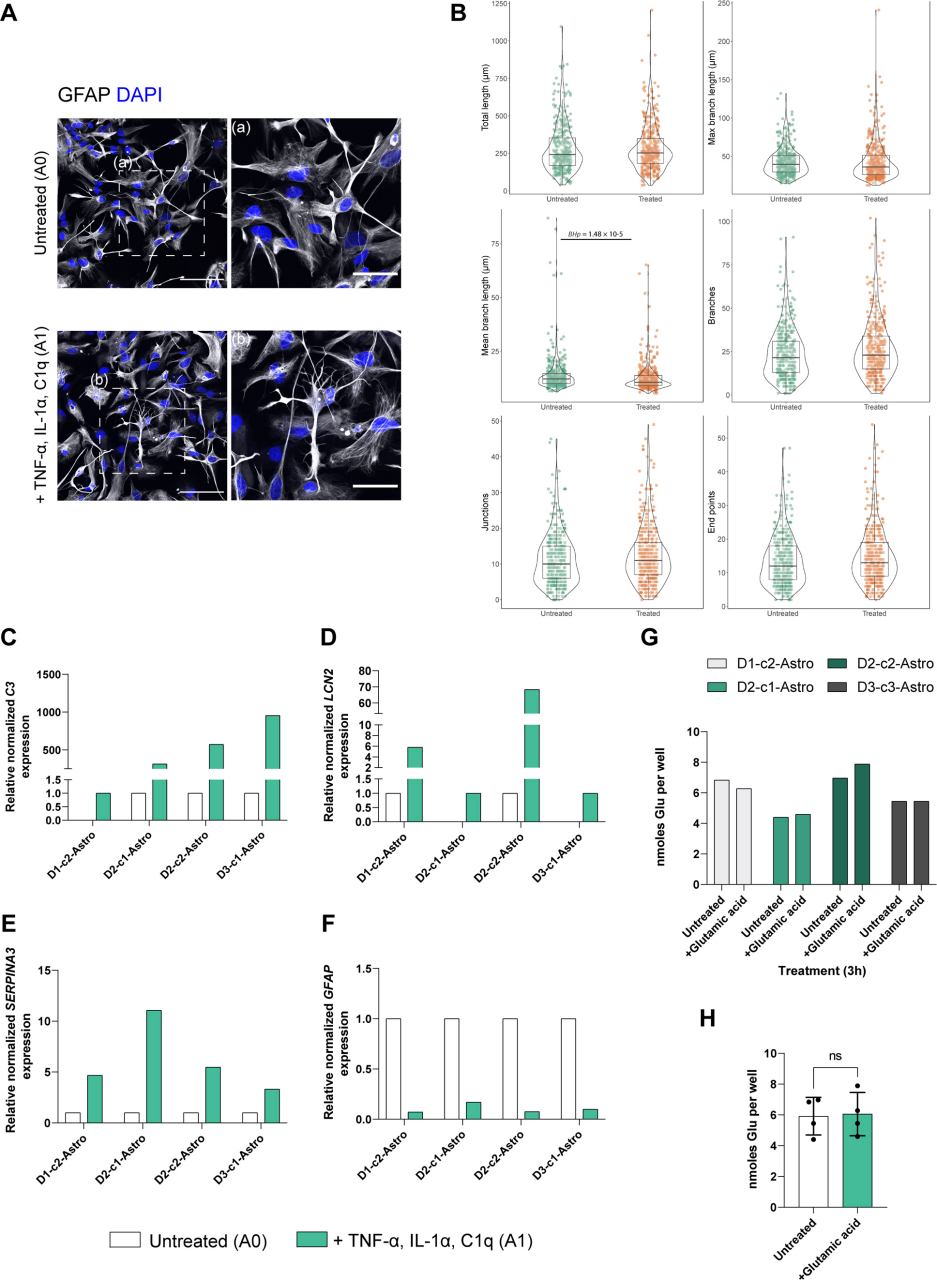
**Changes in astrocyte reactive states and glutamate uptake.** (A) Representative ICC images showing GFAP (gray) expression in astrocytes upon treatment with TNF-α, IL-1α and C1q. Nuclei were counterstained with DAPI (blue). White dashed boxes indicate the magnified images (a,b) to highlight changes in morphology. Scale bars: 100 μm (left); 50 μm (right). Images are representative of one differentiation. (B) Morphological characteristics of 10-week-old astrocytes from reconstructed skeletons before and after TNF-α, IL-1α and C1q treatment. Total length, max branch length and mean branch length are shown in micrometers; the numbers of branches, junctions and end points are reported as counts. A total of 753 skeletons were constructed. (C-F) In 20-week-old astrocytes, reactive astrocyte-markers *C3* (C), *LCN2* (D), *SERPINA3* (E) and *GFAP* (F) were upregulated upon TNF- α, IL-1α, and C1q treatment. Bar charts depict RT-qPCR analysis. Data represent the mean of relative normalized mRNA expression from three technical replicates of one differentiation per line. *CLK2*, *COPS5* and *RNF10* were used as housekeeping genes. (G) Glutamate uptake analysis on 20-week-old astrocytes. Bar graphs show nanomoles of glutamate taken up by each astrocyte line after incubation with 100 µM glutamate for 3 h compared with untreated wells. Data represent the mean amount from two technical replicates of one differentiation per line. (H) Pooled results from G. Each dot represents an astrocyte line. Data show the mean±s.d. Unpaired two-tailed *t*-test; ns, not significant.

Reactive astrocytes play an important role in the pathogenesis of many neurodegenerative diseases (Escartin et al., 2021). To investigate this, several studies have modeled inflammation-stimulated reactivity in iPSC-derived astrocytes (Barbar et al., 2020; Leng et al., 2022; Perriot et al., 2018; Roybon et al., 2013; Santos et al., 2017; Tchieu et al., 2019; Tcw et al., 2017). We also characterized the transcriptomic profile of our astrocytes stimulated with the pro-inflammatory cytokines tumor necrosis factor α (TNF-α, encoded by *TNF*), interleukin 1α (IL-1α, encoded by *IL1A*) and complement component 1, subcomponent q (C1q), which drive an A1-like reactive state (Liddelow et al., 2017) using qPCR. iPSC-derived astrocytes exhibited morphological changes upon cytokine treatment, including the remodeling of GFAP-positive intermediate filaments (Fig. 3A). A proportion of treated astrocytes appeared to have more cellular processes containing GFAP intermediate filaments and more intermediate filament branches than the untreated astrocytes (Fig. 3A, boxed areas).

To quantitatively investigate the changes induced by cytokine treatment on astrocytes, we performed the same morphological analysis we previously employed (Fig. 1F). We characterized the morphological features of 366 untreated and 387 cytokine-stimulated astrocytes. Then, we compared their morphological features with the Mann–Whitney *U*-test (Fig. 3B). The cytokine-stimulated astrocytes showed a significantly shorter mean branch length (*BHp*-value=1.48×10^−5^), whereas the other metrics did not statistically differ across the two conditions in our experimental settings.

Next, we assessed the expression of *C3*, *LCN2*, *SERPINA3* and *GFAP* transcripts, which were affected in astrocytes *in vivo* and *in vitro* upon A1 treatment (Barbar et al., 2020; Liddelow et al., 2017). TNF-α-, IL-1α- and C1q-treated astrocytes showed strong upregulation of *C3*, *LCN2* and *SERPINA3* mRNA transcripts as assessed by RT-qPCR (Fig. 3C-F). The levels of upregulation of *C3*, *LCN2* and *SERPINA3* showed heterogeneous responses across the different astrocyte lines. These differences can be possibly driven by the differences in the phenotypic variation between lines, such as transcriptional pluripotency profiles (Fig. S1B-E). Strikingly, *GFAP* expression was downregulated in the treated astrocytes across all cell lines (Fig. 3F). This contrasts with the changes in the astrocytic cytoskeleton and hypertrophy we detected with GFAP ICC in the treated astrocytes. Overall, we observed similar expression patterns to previous *in vitro* and *in vivo* studies (Barbar et al., 2020; Liddelow et al., 2017). These results show that the generated astrocytes are immunocompetent and respond to inflammatory stimuli by changing their morphology and transcript expression.

We next tested another important astrocyte physiological function, the uptake of glutamate, which prevents neuronal excitotoxicity *in vivo* (Verkhratsky and Nedergaard, 2018). All lines presented detectable levels of intracellular glutamate in baseline conditions (average 5±1 nM, mean±s.d.). Astrocyte lines did not show significant glutamate uptake upon 3 h treatment (average 6±1 nM) (Fig. 3G,H).

### Fluoro-4-AM imaging confirms electrophysiological responsiveness of astrocytes and deCLUTTER^2+^ pipeline reveals dominant profiles and clusters in calcium traces

Neurotransmitter-induced intracellular Ca^2+^ transients play a pivotal role in astrocyte functionality and have been observed in *in vivo* and *ex vivo* systems (Gorzo and Gordon, 2022; Lia et al., 2021). We thus wanted to assess whether the generated astrocytes possessed a similar physiological property.

**Fig. 4.**
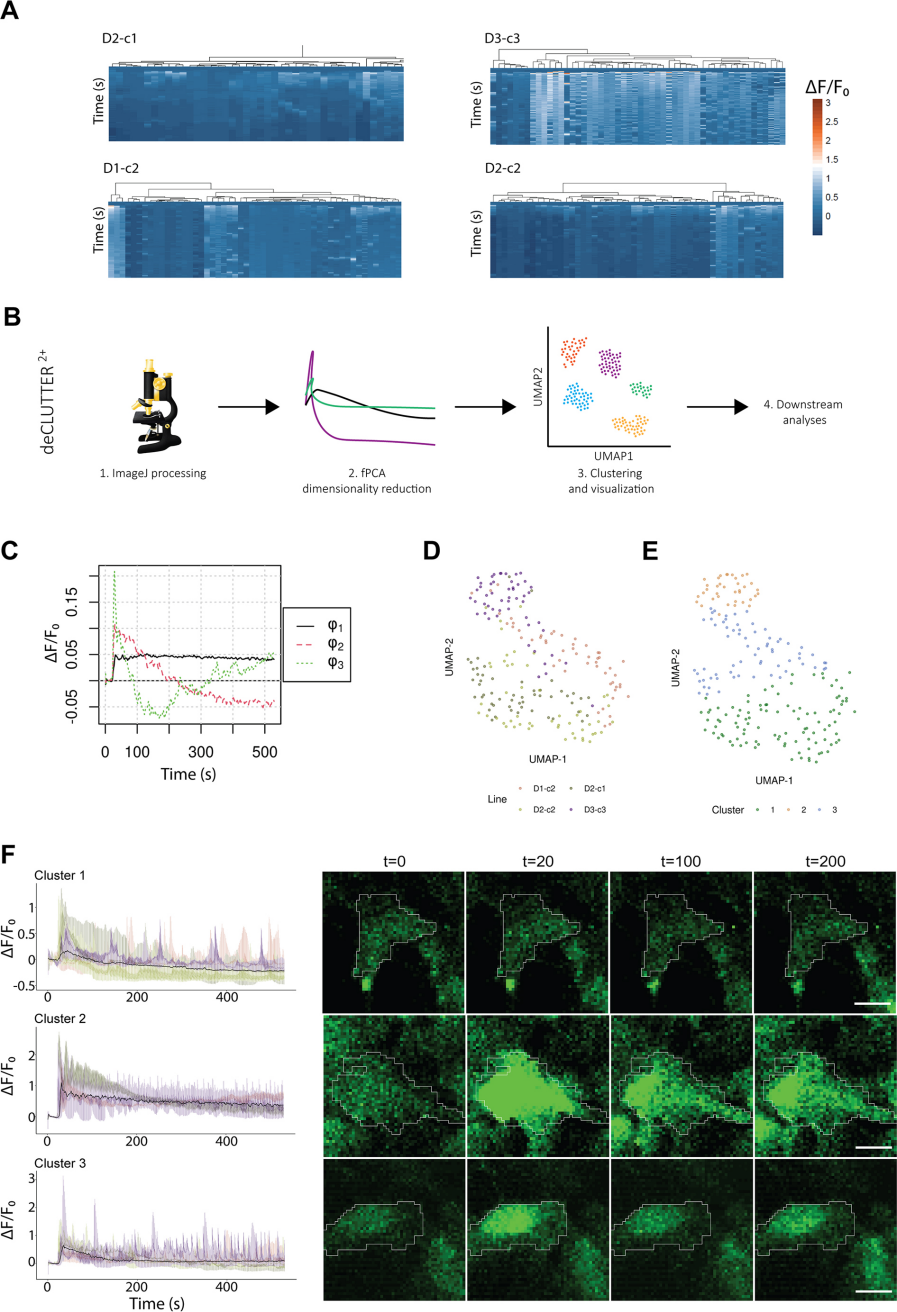
**Characterization of ATP-induced Ca^2+^ transients in astrocytes.** (A) Heatmaps showing normalized Δ*F*/*F*_0_ values for the randomly selected cells for each cell line (*n*=52). (B) Schematic overview of the deCLUTTER^2+^ pipeline. (C) fPCA top three eigenfunctions extracted from Δ*F*/*F*_0_ across the cell lines. (D) UMAP plot showing astrocyte line spread. (E) UMAP plot showing clustering of the cells into the three k-means clusters. (F) Δ*F*/*F*_0_ profiles of the three defined clusters along the imaging time course. Tracks are colored by cell line, and the median Δ*F*/*F*_0_ is highlighted in black. Representative images of the three clusters sampled at time points t=0, 20, 100 and 200 s. Scale bars: 10 μm.

We used Fluo-4 acetoxymethyl ester (Fluo-4-AM) cell loading coupled with ATP stimulation to analyze calcium dynamics in the astrocyte lines. We imaged all the cell lines and identified green-fluorescent regions of interest (ROIs) in the somata using Fiji. We selected 52 cells per line, resulting in a total of 312 cells across six cell lines. Heatmaps showed that the different cell lines had a heterogeneous response to the ATP stimulus, with a variable number of responsive cells. These cells were characterized by distinct patterns with an average peak amplitude of 0.85±0.58 Δ*F*/*F*_0_ (see Materials and Methods for definitions of Δ*F* and *F*_0_) (Fig. 4A). The average peak amplitude was 0.53±0.41 Δ*F*/*F*_0_ for D1-c2 astrocytes, 0.68±0.51 Δ*F*/*F*_0_ for D2-c1 astrocytes, 0.78±0.44 Δ*F*/*F*_0_ for D2-c2 astrocytes and 1.40±0.54 Δ*F*/*F*_0_ for D3-c3 astrocytes. Although to different extents in the number of responsive cells, an increase in fluorescence upon the ATP stimulus was evident in all cell lines.

To further characterize the ATP response behavior, we developed a novel pipeline we named deCLUTTER^2+^ that takes as input the Fluo-4-AM signal calculated with Fiji across the cells. Briefly, functional PCA (fPCA) is applied to perform dimension reduction and denoising (or deCLUTTER^2+^) the signals. Next, cell clusters are identified using k-means. Finally, uniform manifold approximation and projection (UMAP) is applied for visualization of the clusters (Fig. 4B).

Specifically, we applied fPCA to extract the main eigenfunctions (ϕ_n_) describing the dominant patterns of variability across cell lines. The top three eigenfunctions explained 84% of the variability in the Ca^2+^ traces (Fig. 4C). ϕ_1_ (73% of the variability) captured part of the stimulus-associated increase in fluorescence, and then it remained nearly constant with just a slow decrease. ϕ_2_ (8.6% of the variability) encoded the sharp variation in fluorescence around the ATP stimulus time point in responsive cells. Finally, ϕ_3_ (2.7% of the variability) captured both the variability around the ATP stimulus time point and another increase in fluorescence at about halfway through the imaging time course. To show the local and global structures in the cell lines with a two-dimensional representation, we constructed a lower dimensionality embedding with UMAP. We selected the top 19 eigenfunctions, explaining 95% of the variance in the Fluo-4-AM tracks. The different cell lines were admixed onto the UMAP plot, showing a continuum with regions of higher density (Fig. 4D). In the case of the D2-derived lines, it was possible to see an overlap that might be partially driven by a donor effect. We clustered the cell lines using k-means clustering and obtained three distinct groups with a differential contribution from the various cell lines (Fig. 4E). The three clusters were characterized by different Δ*F*/*F*_0_ profiles. The cell lines contributed a variable number of cells to each cluster (Fig. 4F). Clusters 1 and 3 corresponded to low and non-responsive cells, whereas cluster 2 contained cells highly responsive to the ATP stimulus. In addition, deCLUTTER^2+^ showed the variability of the calcium responses at various time points (13, 20 and 30 weeks), with older astrocytes contributing to the clusters of more responsive cells (Fig. S8).

## DISCUSSION

iPSC-derived modeling is gaining momentum as a complementary method to study the properties of human-derived cells under physiological and pathological conditions. However, specific protocols for the generation of iPSC-derived ventral midbrain astrocytes to study the potential role of astrocytes in the non-cell-autonomous neurodegeneration observed in PD have been lagging, and their morphological and functional characterization are limited (Table S1). In our study, we addressed some of these limitations and generate midbrain-specified astrocytes by exploiting developmental events required for astrogenesis.

Recent data indicate that ventral midbrain astrocytes differ from telencephalic astrocytes at the physiological and transcriptomic levels (Siletti et al., 2022 preprint; Xin et al., 2019). However, what could be driving these differences remains to be investigated. Several studies have proposed that such regional heterogeneity of astrocytes arises early in development. The patterning along the neuraxis by sonic hedgehog (SHH), fibroblast growth factors (FGFs), WNTs and bone morphogenic factors (BMPs) leads to a rostral-caudal and dorsal-ventral segmentation of the neuroepithelium into domains that give rise to distinct subtypes of progenitors encoding regional information (Hochstim et al., 2008; Rowitch and Kriegstein, 2010; Sardar et al., 2020; Tsai et al., 2012). Studies performed in various brain regions have demonstrated that astrocytes and neurons derived from a common progenitor show a shared region-specific molecular signature, which might act as a code for region-specific interactions (Herrero-Navarro et al., 2021). Moreover, astrocytes and neurons derived from a common progenitor domain migrate radially and share their final position (Herrero-Navarro et al., 2021; Torigoe et al., 2015). Hence, leveraging the same progenitor pool for the generation of astrocytes and neurons *in vitro* might enable studying neuron-astrocyte interactions of cells carrying a region-specific profile.

Although the developmental trajectory of the ventral midbrain astrocytes is poorly defined, we speculated that the early patterning toward the ventral neural tube step would partially recapitulate the ventral midbrain ontogeny. Therefore, we leveraged well-described patterning protocols using morphogens that specify the ventral neural tube identity (Reinhardt et al., 2013). Importantly, the generated progenitor pool can also differentiate into vmDANs (Grochowska et al., 2021; Reinhardt et al., 2013; Vanhauwaert et al., 2017). Our protocol resulted in a highly pure population of cells expressing classical astrocyte markers. Furthermore, our astrocytes showed strong transcriptomic evidence of the expected differentiation and expressed mature astrocyte markers, including AQP4, GFAP and connexin 43 (GJA1). However, the low expression of some fetal astrocyte markers and the inefficient extracellular glutamate uptake suggest that subpopulations of lesser differentiated astrocytes co-exist with more mature ones. On the other hand, the results of the glutamate uptake assay might have been influenced by the presence of glutamate in the baseline culturing medium, warranting caution in the interpretation of the data. The ratio of mature to immature cells might be further increased using maturation-accelerating supplements, such as the ones reported in a recent preprint for iPSC-derived neurons (Hergenreder et al., 2022 preprint).

Our astrocytes expressed high levels of GFAP, in keeping with its high expression in subcortical regions, whereas lower expression is reported in the cortical regions and the cerebellum (Siletti et al., 2022 preprint; Torres-Platas et al., 2016). However, the absence of a specific midbrain-astrocyte marker makes it challenging to accurately validate the ontogeny of the generated cell line. Single-cell or single-nucleus RNA sequencing analyses have been used to assess astrocyte heterogeneity. However, most of these studies have limitations, including the low yield of isolated astrocytes, which have prevented confidently defining the regional heterogeneity of astrocytes. Recently, high-throughput snRNA sequencing by Siletti et al. (2022 preprint) (three million nuclei sampled) demonstrated multiple astrocyte clusters across the brain and annotated the most variable genes across different astrocyte populations. By re-analyzing their datasets from astrocytes dissociated from the adult midbrain, cortex and hippocampus, we first generated a list of candidate markers. Then, we evaluated their expression in our astrocyte cultures. Interestingly, our candidate markers contained several genes previously linked to region-specific expression and function. For example, *HGF* and *HSPB3* are expressed in cortical regions, whereas *PITX2* and *NKX6-1* are linked to midbrain development (Martin et al., 2004; Prakash et al., 2009). Overall, these data suggest that astrocytes generated by our protocol display characteristics of *in vivo* midbrain astrocytes.

As chromosomal abnormalities are known and recurrent in iPSC cultures, affirming a normal karyotype has become a fundamental quality control step prior to differentiation. However, this is not a widely adopted procedure for cells differentiated from iPSCs, including astrocytes generated with published protocols. Importantly, our study shows how chromosomal aberrations can be introduced during iPSC differentiation procedures, warranting for karyotype validation at various differentiation steps. This is a critical aspect to ensure that the effects observed in the downstream analyses are not due to the chromosomal abnormalities in some of the lines. Unfortunately, we do not know how common this is in other differentiation protocols, but it might be a reflection point for the formulation of highly needed guidelines for the field. A general agreement on the best practices for culturing, differentiation and validations of iPSC-derived cells is necessary to promote robustness of the results.

Next, we aimed to generate a model that recapitulates some of the properties of astrocytes. We emphasize our findings from these functional assays, establishing iPSC-derived astrocyte cultures as a platform for disease modeling. Recent research using post-mortem samples identified reactive astrocytes in the midbrain of individuals with PD (Schonhoff et al., 2020). Therefore, we investigated the responses of astrocytes to pro-inflammatory stimuli by treating the cells with TNF-α, IL-1α and C1q, which drive a reactive phenotype (Escartin et al., 2021; Liddelow et al., 2017). This assay has been widely used to study neuroinflammation *in vitro* and *in vivo* (Barbar et al., 2020; Leng et al., 2022; Liddelow et al., 2017; Zhou et al., 2019). Moreover, the morphology and transcriptomic profile of stimulated astrocytes have been well characterized. Our protocol generated astrocytes that were immunocompetent. In particular, we observed a strong upregulation of complement component 3 (C3), also found in the astrocytes residing in the substantia nigra pars compacta of idiopathic PD cases (Liddelow et al., 2017). Moreover, we observed similar expression patterns for *GFAP*, *LCN2* and *SERPINA3* transcripts, which were in line with *in vivo* studies and previously published iPSC-derived astrocyte protocols (Barbar et al., 2020; Liddelow et al., 2017). Altogether, we characterized a cellular *in vitro* model that has the potential to be applied in mechanistic studies on neuroinflammatory signaling in PD.

The effectiveness of our protocol is further supported by the data showing heterogeneous Ca^2+^ transients in ventral midbrain astrocytes upon ATP treatment. These currents are known to have a critical impact on neurotransmitter release (Bezzi and Volterra, 2001), synaptic transmission and plasticity (Di Castro et al., 2011; Panatier et al., 2011), and even behavior and cognition (Kofuji and Araque, 2021; Nagai et al., 2021; Santello et al., 2019), and are widely used as functional readouts *in vivo* and *in vitro* (Gorzo and Gordon, 2022; Lia et al., 2021). Importantly, calcium dysregulation is a potential mechanism for several genes implicated in PD (Zaichick et al., 2017). Abnormal calcium signaling in the astrocytes might cause dysfunction in dopaminergic neurons, activate microglia and disrupt the blood-brain barrier integrity, therefore contributing to the pathological mechanisms seen in PD (Bancroft and Srinivasan, 2021). The classical processing of calcium images from iPSC-derived astrocytes has encompassed visualization of the transients via time-lapse imaging and traces. To better integrate the data from the different cell lines, we developed the novel deCLUTTER^2+^ pipeline. deCLUTTER^2+^ can be used to perform semiautomatic recognition of spontaneous or cue-dependent recurring patterns in fluorescence time-lapse imaging. Our approach can handle variability and can be used to easily visualize local and global structures among the analyzed cells. We anticipate that this approach could be used in the context of disease modeling to study the association of specific cluster(s) to conditions.

In conclusion, we generated ventral midbrain patterned astrocytes that express midbrain-specific and astrocyte-specific markers and recapitulate some of the physiological properties of *in vivo* ventral midbrain astrocytes. Our ventral midbrain astrocyte model can also be integrated with neurons and other glial cell types, going beyond traditional two-dimensional co-culture systems (Majumdar et al., 2011; Park et al., 2018). Interestingly, we observed an increase in the expression of genes that play essential roles in synaptogenesis (*SPARCL1*) and neurotransmitter recycling (*SLC1A3* and *SLC1A4*), indicating potential neurotrophic properties of astrocytes generated by our protocol. Future research will investigate this possibility in more detail. Moreover, such neuron-glia interaction paradigms might help elucidate the pathological processes observed in neurodegenerative diseases, such as PD, and potentially pave the way toward disease-modifying treatments.

## MATERIALS AND METHODS

### Primary cell lines

We generated eight human iPSC lines (Table S4). Clonal lines D1-c1-iPSC and D1-c2-iPSC were generated from the commercially available dermal fibroblasts from a female donor (Gibco, lot number 1903939). The skin biopsy was sampled at the age of 33. Clonal lines D2-c1-iPSC and D2-c2-iPSC were derived from the commercially available dermal fibroblasts from a female donor (Gibco, lot number 181388). The skin biopsy was sampled at the age of 34. Clonal lines D3-c1-iPSC, D3-c2-iPSC, D3-c3-iPSC were generated from the primary fibroblasts from a female donor that were available in our institute. The skin biopsy was sampled at the age of 68. Line D4-c1-iPSC was derived from the primary cultures of erythroid progenitors (Leberbauer et al., 2005; van den Akker et al., 2010), which were generated from the peripheral blood from a female donor that was available in our institute. The blood was sampled at the age of 83. All study procedures were approved by the medical ethical committee of Erasmus MC and conformed to the principles of the World Medical Association (WMA) Declaration of Helsinki and the Department of Health and Human Services Belmont report. Participating subjects provided written informed consent for the use of the material for research purposes.

### Generation of iPSC lines

Reprogramming of all primary cell lines into iPSCs used in this study was performed by the Erasmus MC iPS Core Facility. Primary cell lines were reprogrammed using the CytoTune-iPS 2.0 Sendai Reprogramming Kit (Thermo Fisher Scientific) based on a modified non-transmissible form of Sendai virus (SeV), which contains reprogramming Yamanaka factors, OCT3/4, SOX2, KLF4 and C-MYC. The emerging iPSC colonies were manually picked and expanded for 4-5 weeks after transduction. The selection was based on morphology. iPSCs were cultured in StemFlex medium (Gibco) on Geltrex (Thermo Fisher Scientific)-coated plates at 37°C and 5% CO_2_. iPSCs were passaged when they reached ∼80% confluence using Versene (Gibco). Multiple clones per line were assessed for their karyotype, expression of endogenous pluripotency factors (RT-qPCR and ICC) and differentiation potential into three lineages (ectoderm, endoderm and mesoderm) following the procedures of the Erasmus MC iPS Core Facility. The human embryonic stem cell lines (HUES1, HUES2, HUES6 and HUES9) used in the RT-qPCR experiments (Fig. S1) were a kind gift from Chad A. Cowan (Harvard University, Harvard Stem Cell Institute).

### Primary and secondary antibodies

The primary antibodies used in this study are listed in Table S3. Alexa Fluor 488 donkey anti-goat/mouse/rabbit (AB_2340428, AB_2340846 and AB_2313584), Alexa Fluor 594 donkey anti-mouse/rabbit (AB_2340854 and AB_2340621), Alexa Fluor 647 donkey anti-rabbit (AB_2492288) and Alexa Fluor 647 goat anti-guinea pig (AB_2337446) (all from Jackson ImmunoResearch) were used as secondary antibodies for ICC experiments and microscopic analysis.

### Characterization of iPSC lines with ICC

iPSCs grown on coverslips were fixed with 4% paraformaldehyde for 15 min at room temperature (RT). Cells were washed three times for 5 min with 1× PBS. Next, cells were incubated with ice-cold methanol for 10 min and washed one time with 1× PBS. Subsequently, cells were permeabilized with 0.1% Triton X-100 (Sigma-Aldrich) in 1× PBS for 10 min. Cells were blocked in a blocking buffer containing 1% bovine serum albumin (Sigma-Aldrich) in 1× PBS containing 0.05% Tween 20 (Sigma-Aldrich). Then, cells were incubated with the blocking buffer containing primary antibody mixtures overnight at 4°C. The next day, cells were washed three times for 5 min in 1× PBS containing 0.05% Tween 20. Cells were then incubated with appropriate Alexa Fluor-conjugated secondary antibodies (1:500 dilution) for 1 h at RT. Cells were washed three times with 1× PBS containing 0.05% Tween 20. Finally, coverslips were mounted with ProLong Gold with DAPI (Invitrogen). All stained samples were imaged with a Leica SP5 AOBS confocal microscope. Each image was detected on the spectral photomultiplier tube (PMT) detector with an HCX PL APO CS 40×/1.25 or HCX PL APO CS 63×/1.4 lens. Sections were irradiated with the following lasers: 405 diode UV, argon laser, DPSS 561 and HeNe 633, depending on the fluorophore combination. Scanning was performed at 400 Hz with a pixel size of 0.12 µm in the *x-* and *y*-direction and 0.35 µm in the *z*-direction. The pinhole size was set to one airy unit.

### Generation of neural progenitor cells

Neural progenitor cells were generated by inhibiting BMP and TGFβ signaling (dual-SMAD) and stimulation of WNT and SHH signaling with small molecules according to published protocols (Grochowska et al., 2021; Reinhardt et al., 2013) with few modifications. iPSCs (early passages, between 6 and 15) grown in feeder-free conditions (StemFlex in combination with Geltrex) were detached from the plates using the Versene solution. Cells were dissociated as clumps and plated on irradiated mouse embryonic fibroblasts (MEFs) in StemFlex medium supplemented with 1× RevitaCell (Gibco) in a splitting ratio of 1:10. Cells were cultured at 37°C and 5% CO_2_ until the iPSC colonies reached the appropriate size in StemFlex medium. Unwanted differentiations that arose around the iPSC colonies were manually removed. When the colonies populated ∼70% of the culture dish, cells were detached as clumps from the MEFs using Versene. Next, pieces of colonies were resuspended in human embryonic stem cell medium [HESC, 80% Dulbecco's modified Eagle medium (DMEM)/F-12, 20% KnockOut Serum Replacement, 1% L-glutamine, 1% penicillin-streptomycin, 1% minimum essential medium (MEM)-non-essential amino acids solution (NEAA) (all from Gibco) and 0.0007% 2-β-Mercaptoethanol (Sigma-Aldrich)] supplemented with 10 µM SB-431542 (Tocris), 1 µM dorsomorphin (Abcam), 3 µM CHIR99021 (CHIR; Sigma-Aldrich), and 0.5 µM purmorphamine (PMA, Stem Cell Technologies). Clumps of cells were then transferred to 10 cm Petri dishes and cultured in suspension for 6 days on a shaker at 80 rpm at 37°C with 5% CO_2_. On day two, the medium was replaced with N2B27 medium [DMEM/F-12 and neurobasal medium in 1:1 ratio, 1:100 B27 without vitamin A, 1:200 N2 and 1% penicillin-streptomycin (all from Gibco)] supplemented with 10 µM SB-431542, 1 µM dorsomorphin, 3 µM CHIR and 0.5 µM PMA. On day four, the medium was changed to N2B27 medium supplemented with 3 µM CHIR, 0.5 µM PMA and 150 µM ascorbic acid (AA; Sigma-Aldrich). On day six, embryoid bodies showing a developing neuroepithelium were collected, dissociated into smaller pieces, and plated on Corning Matrigel-coated 12-well plates in N2B27 medium supplemented with 3 µM CHIR, 200 µM AA and 0.5 µM PMA at 37°C and 5% CO_2_. Cell splits were usually performed at 1:10 or 1:20 ratios. After passage five, 0.5 µM PMA was replaced by 0.5 µM Smoothened agonist (SAG, Abcam). Progenitors were passaged at least five times before astrocyte differentiations. Progenitors could be expanded in bulk (up to passage 30) and frozen for long-term storage.

### Differentiation of neural progenitors into ventral midbrain patterned astrocytes

Neural progenitors (early passages, 6-10) that reached 70-80% confluence were dissociated into single cells using Accutase (Sigma-Aldrich). Cells were seeded in ultra-low-attachment 96-well U-bottom plates (BIOFLOAT, faCellitate) at a concentration of ∼15,000 cells per well in N2B27 medium supplemented with 3 µM CHIR, 0.5 µM SAG and 200 µM AA. Before placing in the incubator, plates were spun down at 220 ***g*** for 5 min. Cells were cultured at 37°C and 5% CO_2_. After 3 days, progenitor cells formed spheres of sizes around 300-400 µm. On day three, the medium was replaced with a glial expansion medium containing astrocyte basal medium (ABM) [DMEM/F-12, GlutaMAX, 1:50 B27 without vitamin A, 1:100 N2, 1× MEM-NEAA, 1% penicillin-streptomycin (all from Gibco)], freshly supplemented with 10 mM HEPES (Gibco), 10 ng/ml EGF (Peprotech) and FGF-2 (Peprotech). The medium was refreshed every other day. Cells were kept at 37°C and 5% CO_2_. On day 17, the medium was switched to a glial induction medium containing ABM, freshly supplemented with 10 mM HEPES, 10 ng/ml EGF (Peprotech) and 10 ng/ml LIF (Peprotech). The medium was refreshed every other day until day 31. Cells were kept at 37°C and 5% CO_2_. On day 31, 15-20 spheres per line were carefully taken and plated onto one well of a six-well plate coated with Corning Matrigel. Generally, spheres easily attached to the coated surface. After attachment to the coated plates, the cells migrated out of the spheres. From day 31, the cultures were maintained in glial maturation medium containing ABM, freshly supplemented with 10 mM HEPES, and 10 ng/ml CNTF (Peprotech). The medium was refreshed every other day until day 59. From day 60 onwards, cells were refreshed every 2 days with a glial maintenance medium containing ABM, freshly supplemented with 10 mM HEPES. From day 60, cells were used in downstream functional experiments. Cells dissociated with accutase (Fig. 1Be) were plated onto new Matrigel-coated wells at 50,000 cells/cm^2^ when they repopulated the entire well. Detailed cell culture medium composition is listed in Table S2.

### Characterization of iPSC-derived progenitors and astrocytes with ICC

Cells were fixed with 4% paraformaldehyde for 15 min at RT. Cells were washed three times for 5 min with 1× PBS. Next, cells were incubated in a staining buffer [50 mM Tris.HCl (pH 7.4), 0.9% NaCl, 0.25% gelatine, H_2_O] containing primary antibody mixtures overnight at 4°C. The next day, cells were washed three times for 5 min in 1× PBS containing 0.1% Tween 20. Cells were then incubated with appropriate Alexa Fluor secondary antibodies (1:200 dilution) for 1 h at RT. Secondary antibodies were washed three times with 1× PBS containing 0.1% Tween 20. Finally, cells were mounted with ProLong Gold with DAPI (Invitrogen). Each image was detected on the spectral PMT detector with an HCX PL APO CS 40×/1.25 or HCX PL APO CS 63×/1.4 lens. Sections were irradiated with the following lasers: 405 diode UV, argon laser, DPSS 561, and HeNe 633, depending on the fluorophore combination. Scanning was performed at 400 Hz with a pixel size of 0.12 µm in the x, y-direction and 0.35 µm in the z-direction. The pinhole size was set to 1 airy unit. All images were processed using Fiji (Fiji Is Just ImageJ) software (version 1.53c) (Schindelin et al., 2012). For the quantitative characterization of neural progenitors and astrocytes, fluorescence-based thresholding was applied for each marker. The cell was considered positive for a marker if its fluorescence signal was above that threshold and within the boundaries of that cell.

The ImageJ plug-in ‘Skeletonize 2D/3D’ (Arganda-Carreras et al., 2010) was used to perform the quantitative morphological characterization of the astrocytes. To aid in the identification of the cell borders of the astrocyte, we employed a previously described strategy (Schwendy et al., 2019). Briefly, the local maxima of each cell in a *z*-projected image were determined to create an inverted tile mask with one segmented particle (tile) per maximum. Next, the background threshold method as described by Li and Lee (1993) and Li and Tam (1998) was used to select the total cell area in the image and create another mask. ROIs were identified using the ‘Analyze Particle’ plug-in. Each ROI was manually examined and refined using the ‘Selection Brush’ plug-in. Clumps of cells, cells with multiple nuclei, and other artifacts were manually removed. Finally, cell skeletons and their quantitative characteristics were obtained with the plug-in ‘Skeletonize 2D/3D’.

### RNA extraction, sequencing and alignment to the reference genome

RNA was extracted from 14 iPSC-derived lines (eight progenitors and six astrocytes) generated from four individuals (Table S4). Cells were harvested and lysed with lysis buffer (1 ml RNeasy Lysis Buffer+10 μl β-mercaptoethanol) and scraped with a polypropylene disposable cell scraper. Cell lysates were transferred to 1.5 ml tubes and vortexed to dissolve possible cell clumps. Samples were further processed using the QIAGEN RNeasy Mini Kit (GTIN 04053228006121, lot 172019069, reference 74106) according to the manufacturer's instructions. RNA fragments were analyzed using the RNA 6000 Nano Kit on an Agilent 2100 Bioanalyzer instrument (Agilent Technologies) to determine the RNA integrity number (RIN). About 1 µg high-quality RNA sample (average RIN 9.9; Table S4) from each sample was further processed with the Illumina NEBNext Ultra II Directional RNA Library Prep Kit (New England Biolabs, E7760S/L) according to the manufacturer's instructions. The generated libraries were sequenced on an Illumina NovaSeq6000 with 150 bp paired-end reads and an average of 60 million reads per sample (GenomeScan, Leiden, the Netherlands). RNA-seq data quality was assessed using fastqc v0.11.9 (https://www.bioinformatics.babraham.ac.uk/projects/fastqc/) and summarized using MultiQC v1.12 (Ewels et al., 2016). RNA-seq reads were aligned to the GRCh38 human reference genome with STAR v2.7.10 (Dobin et al., 2013) run in multisample two-pass mapping mode to improve the detection of novel splice junctions. The counts of reads per gene were determined using FeatureCounts v2 (Liao et al., 2014) using ENSEMBL gene annotations v107 (https://ftp.ensembl.org/pub/release-107/).

### Cell line karyotyping

RNA-seq data were processed according to the Genome Analysis Toolkit (GATK) v4.2.5 guidelines (McKenna et al., 2010) to generate a variant call format (VCF) with the ‘HaplotypeCaller’ tool. The possible presence of chromosomal multiplications was then assessed using eSNP-karyotyping (Weissbein et al., 2016) with the binary alignment map (BAM) and VCF in combination with dbSNP v155 (https://www.ncbi.nlm.nih.gov/snp/). As previously described, the allelic ratio in sliding windows of 151 single-nucleotide polymorphisms (SNPs) with a depth minimal allele frequency above 0.2 and covered by more than 20 reads was compared with that of the rest of the genome. Significant multiplications were identified as those with a false discovery rate <0.05.

We performed high-resolution SNP array (SNP-A) karyotyping on genomic DNA extracted from the progenitor lines using the Infinium Global Screening Array-24 v3.0 kit (Illumina, San Diego, CA, USA). Genotyping data were than analyzed in Nexus 10 (BioDiscovery) to detect chromosomal abnormalities.

### RNA-seq expression data analyses

Digital expression matrices of the analyzed cell lines were analyzed in R v4.2.1. Established cell-type-specific markers were obtained from BRETIGEA v.1.0.3 (McKenzie et al., 2018). Moreover, we used previously described markers to characterize precursor cells and the maturity of the astrocytes (Sloan et al., 2017). Low-expressed genes (<10 cumulative raw counts across all samples) were filtered out, resulting in 27,761 expressed genes. DESeq2 v1.36.0 (Love et al., 2014) was used to transform raw counts, assess sample-to-sample distances and clustering via PCA on rlog-transformed data, and perform differential expression analysis. The obtained *P*-values were corrected with the Benjamini–Hochberg (BH) method (*BHp*-value), and DEGs were identified according to the following thresholds: *BHp*-value <0.05 and |fold change (FC)| >1.5.

### RNA-seq functional analysis

GSEA was performed using the clusterProfiler v4.4.4 (Wu et al., 2021) and org.Hs.eg.db v3.15.0 (https://bioconductor.org/packages/release/data/annotation/html/org.Hs.eg.db.html) R packages with the Gene Ontology (GO) database. GO terms were filtered to include sets consisting of 20-2000 genes. The genes in our datasets were ranked in descending order by the negative logarithm in base 10 of the adjusted *BHp*-value, multiplied for the sign of the fold change. All resulting *P*-values were corrected using the BH method. A nervous system-centric analysis was conducted by extracting the 181 terms surviving our filtering criteria out of the 1256 child terms of the ‘nervous system development’ set (GO:0007399).

### Identification of candidate midbrain astrocyte markers

Despite the reported regional heterogeneity of the astrocytes in the human brain (Batiuk et al., 2020; Siletti et al., 2022 preprint), there is no consensus on midbrain-specific markers for astrocytes. To further characterize the regionalization of our astrocytes, we aimed to identify midbrain-specific markers using recently published single nucleus RNA-sequencing (snRNA-seq) data from Siletti et al. (2022 preprint). Using loompy v3.0.6 (https://loompy.org/), we selected the astrocyte clusters from three brain regions (the midbrain, the cerebral cortex and the hippocampus) as they contain physiologically distinct astrocyte populations (Xin et al., 2019). Next, we generated pseudo-bulk datasets by summing individual cell gene expression values within each donor and brain region. Finally, we tested for differences in gene expression among the region-pooled astrocytes using DESEq2 v1.36.0 (Love et al., 2014). Among the significantly differentially expressed genes (*BHp*-value<0.05), we selected the top ten genes (ordered by decreasing FC value) that were also expressed in our cell lines.

### Pro-inflammatory cytokine treatment – A1-like reactivity assay

We adapted the reactivity assay from Barbar et al. (2020) to obtain an A1 reactive astrocyte phenotype. Astrocytes were plated onto new Matrigel-coated wells at 50,000 cells/cm^2^. Two days later, astrocytes were treated with 30 ng/ml TNF-α, 3 ng/ml IL-1α and 400 ng/ml C1q for 24 h. After 24 h, cells were collected, and the total RNA was isolated using the RNeasy Mini Kit (QIAGEN) as recommended by the manufacturer. For each sample, on-membrane DNase I (RNase-Free DNase Set, QIAGEN) digestion was performed according to the manufacturer's protocol. The integrity of the total RNA was assessed using agarose gels stained with GelRed (Biotium) and spectrophotometric analysis using a NanoDrop 2000/2000c (Thermo Fisher Scientific). Sharp, clear 28S and 18S rRNA bands without smearing and A_260/280_ values of ∼2.0 indicated intact and pure RNA. Next, 0.5 µg of the total RNA was converted into cDNA using the iScript cDNA Synthesis Kit (Bio-Rad). qPCR was performed on a CFX Opus 96 Real-Time PCR System (Bio-Rad) with ∼0.1 µg cDNA per reaction. The following cycling conditions were used: 3 min at 95°C (initial denaturation), 40 cycles of 5 s at 95°C, and 30 s at 60°C. Data analysis was performed using CFX Maestro software 2.3 (Bio-Rad). Briefly, the normalized expression of each target gene was calculated using the ΔΔCq method (Livak and Schmittgen, 2001). Relative mRNA levels for *C3*, *LCN2*, *SERPINA3* and *GFAP* were determined after normalization to the geometric mean of the mRNA levels of the following housekeeping genes: *COPS5*, *CLK2* and *RNF10*. All assays, spanning at least one intron, were validated by demonstrating linearity over three orders of magnitude and by observation of a single melt peak by plotting relative fluorescence unit (RFU) data collected during a melt curve as a function of temperature. Primers were adapted from previously published articles or designed using the Primer3 v. 0.4.0 online tool (https://bioinfo.ut.ee/primer3-0.4.0/) and are listed in Table S5.

### Glutamate uptake assay

Astrocytes grown in six-well plates were refreshed with glial maintenance medium or glial maintenance medium supplemented with 100 µM L-glutamic Acid (Tocris) for 3 h. The glutamate concentration in astrocyte cultures was determined using the glutamate assay kit (Sigma-Aldrich, MAK004). To obtain cell homogenates, astrocyte cultures were lifted with Accutase, briefly centrifuged at 220 ***g*** for 5 min, and resuspended with 100 μl of the glutamate assay buffer. Samples were then centrifuged at 13,000 ***g*** for 10 min to remove insoluble material. Enzymatic reaction mixes were prepared by mixing glutamate assay buffer, developer and enzyme mix according to the manufacturer's specifications and were subsequently mixed with 50 μl of cell homogenates and incubated for 30 min at 37°C. Absorbance was measured at a 450 nm wavelength in a Varioskan microtiter plate reader (Thermo Fisher Scientific). Samples were run in duplicate and compared with the glutamate standard after subtracting the blank lacking the enzyme mix.

### Intracellular Ca^2+^ imaging

Two days before live-cell imaging, confluent astrocytes were seeded on Matrigel-coated 24-mm round glass coverslips in a 1:3 ratio. On the day of imaging, cells were treated with a loading solution of DMEM/F-12 without Phenol Red (Gibco) containing 2 µM cell-permeant Fluo-4-AM (Thermo Fisher Scientific) with the addition of an equal quantity (1:1 v/v) of 20% Pluronic F-127 (Thermo Fisher Scientific) to assist in the dispersion of the nonpolar Fluo-4-AM ester in the aqueous medium. After incubation with the loading solution for 30 min at 37°C, the astrocytes were washed thoroughly three times with DMEM/F-12 without Phenol Red to remove any dye non-specifically associated with the cell surface. Finally, coverslips were mounted in live-cell imaging chambers and immediately transferred to a Leica SP5 AOBS inverted confocal microscope equipped with a live-cell module maintaining a 37°C, 5% CO_2_ and >90% relative humidity environment. After a 1-min time-lapse series acquisition, ATP solution (Sigma-Aldrich) was added to the medium. The final concentration of ATP in the medium was 100 μM. The ATP was added carefully using a conventional pipette, without touching the imaging chamber, to avoid movement artifacts and to ensure that the same field of view was imaged before and after the ATP application. Finally, a 10-min time-lapse series was acquired to record the ATP-induced fluorescence. Series were recorded at 400 Hz with 1 fps acquisition. Each frame was detected on a spectral PMT detector with an HCX PL APO CS 20×/0.7 DRY UV lens. Cells were irradiated with the argon laser.

### Quantification of calcium imaging traces with deCLUTTER^2+^ pipeline

All images were processed using Fiji software (version 1.53c). Raw time-lapse imaging stacks (*x*, *y*, *t*) of intracellular Ca^2+^ were first corrected for drift using the ‘Correct 3D Drift’ plug-in (Parslow et al., 2014). To obtain individual Ca^2+^ traces, cells were segmented with a semi-automated segmentation strategy adapted from a method described by Schwendy et al. (2019). To estimate cell borders, the local maxima (mainly located in the cell nucleus) of each cell in a *z*-projected image were determined to create an inverted tile mask with one segmented particle (tile) per maximum. Next, another mask was made using a Li background threshold method (Li and Lee, 1993; Li and Tam, 1998) to select the total cell area in the image. In this case, the ‘leaky’ fluorescence from the Fluo-4-AM was exploited to identify all the cell bodies via a thresholding method. A logical ‘XOR’ operation using the ‘Image Calculator’ was performed on both masks. To smooth the objects and remove isolated pixels, erosion and dilation were performed using the ‘Open’ function in the Binary submenu. The remaining holes were filled using the ‘Fill Holes’ function in the Binary submenu. ROIs were created using the ‘Analyze Particle’ plug-in. Each ROI was manually inspected to ensure that it defines only one cell. ROIs connected at one or two pixels were separated with ‘Selection Brush’. Lastly, mean gray values per time frame were calculated in each ROI. For each time point in each ROI, we calculated the signal-to-baseline ratio of fluorescence *F* using the formula 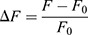, where the baseline *F*_0_ is estimated as the average of the fluorescence levels of 20 time points before ATP addition. The traces were visualized using the ggplot2 v3.3.6 (https://ggplot2.tidyverse.org/) and pheatmap v1.0.12 (https://cran.r-project.org/web/packages/pheatmap/) R packages.

To capture the time functions underlying the calcium dynamics observed in the astrocytes, we employed fPCA. With this technique, it is possible to analyze a set of observations ordered in time, i.e. functions, and to identify the underlying eigenfunctions (ϕ) that describe the shape of the data. Similarly to the principal components in PCA, eigenfunctions are ranked by the amount of variance they explain and can be used to reduce the dimensionality of the dataset. Δ*F*/*F*_0_ values from the six cell lines were merged and analyzed with the fdapace v0.5.9 (Wang et al., 2016) R package. Next, we selected the top eigenfunctions explaining 95% of the variance in the data. We used them as an input for UMAP onto two dimensions using the umap v0.2.9.0 R package (https://cran.r-project.org/web/packages/umap/). Finally, we performed k-means clustering using the factoextra v1.0.7 0 R package (https://cran.r-project.org/web/packages/factoextra/) and selected k=3 using the elbow plot method and visual evaluation of the UMAP plot.

### Statistical analyses

Statistical analyses were carried out using GraphPad Prism 9 (San Diego, CA, USA). Unpaired two-tailed *t*-test was performed for the analysis of the glutamate uptake assay. Two-way ANOVA with Tukey’s post hoc test was applied to the ICC data of astrocytes. Effects with *P*-value <0.05 were considered significant.

## Supplementary Material

10.1242/dmm.049980_sup1 Supplementary information
