# Supplementary material for: deCLUTTER2+ – a pipeline to analyze calcium traces in a stem cell model for ventral midbrain patterned astrocytes
Source: Dis Model Mech. 2023 Jun 23;16(6):dmm049980. doi: 10.1242/dmm.049980 (PMC10309582; doi:10.1242/dmm.049980)
Supplement: Supplementary information [file dmm-16-049980-s1.pdf]

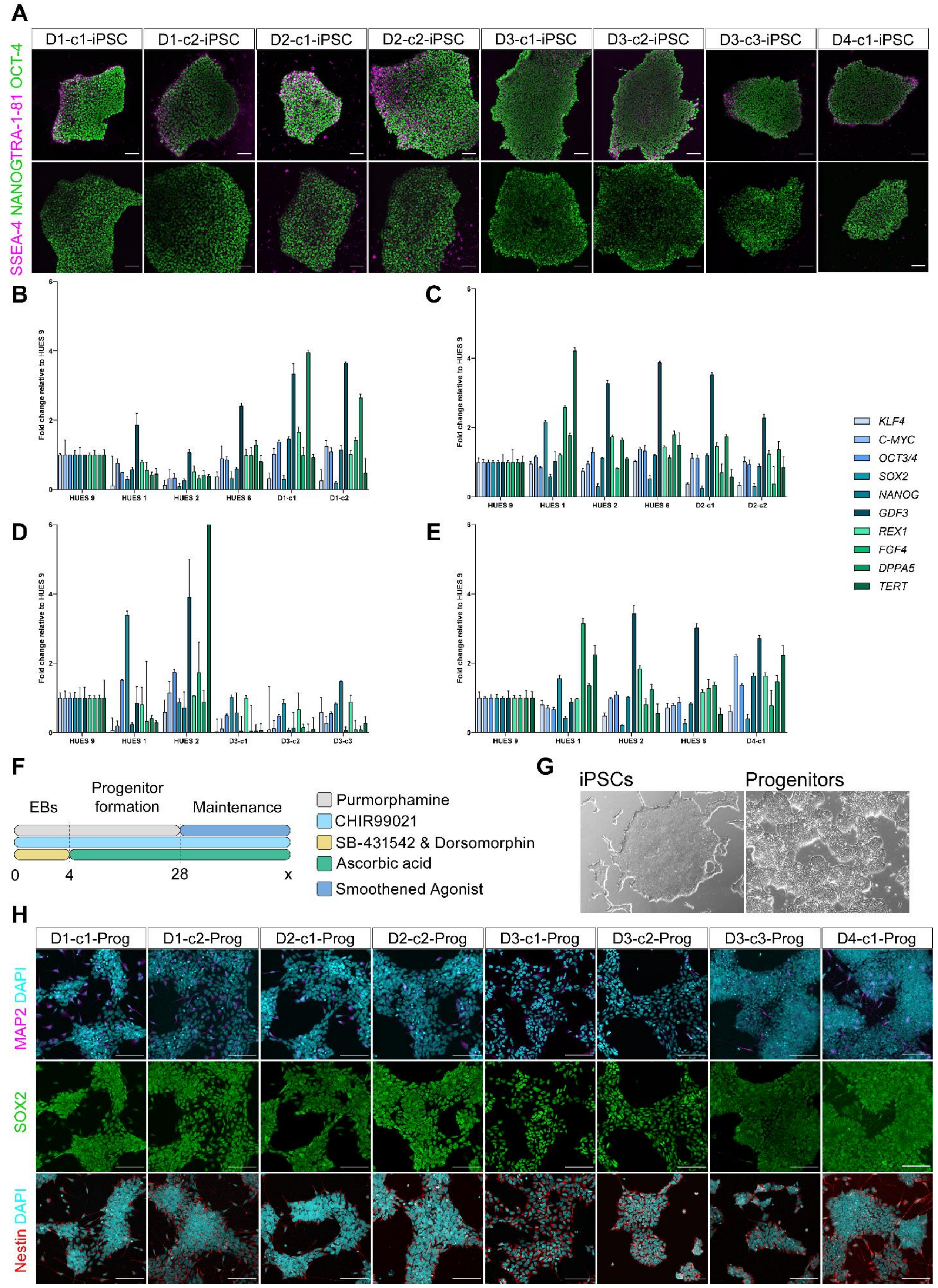

Disease Models & Mechanisms • Supplementary information

Fig. S1. **Generation and characterization of iPSCs and iPSC-derived ventral neural tube progenitors.** A. Representative ICC images of eight iPSC lines staining positive for general pluripotency markers OCT4, TRA-1-81, NANOG, and SSEA-4. **B-E.** Expression analysis by RT-qPCR of pluripotency markers in the generated iPSC lines. The expression was normalized to the reference line HUES9. **F**. Schematic of the progenitor differentiation protocol depicting the major steps with the accompanying supplements. **G**. Representative bright field images of feeder-free iPSC cultures and progenitor cultures. **H.** Representative ICC images from three independent stainings of eight iPSC-derived ventral neural tube patterned progenitors staining positive for neural progenitor markers SOX2 and Nestin and negative for mature neuron marker MAP2. Scale bars, 50 µm (in **C**) or 100 µm (in **D**). Nuclei were counterstained with DAPI (cyan)

Disease Models & Mechanisms • Supplementary information

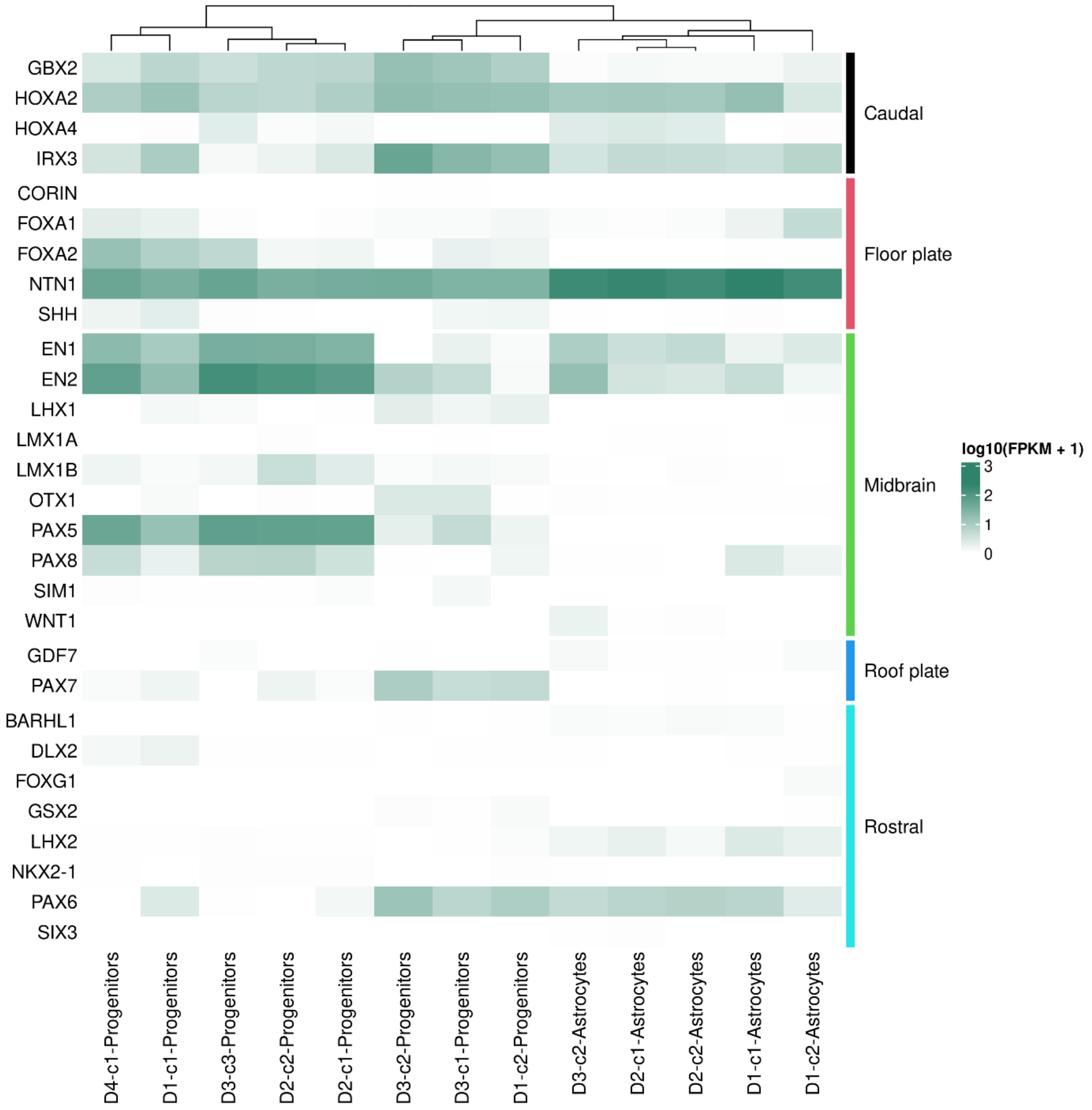

Fig. S2. RNA-seq analysis of eight progenitor lines and six astrocyte lines showing expression of genes that are markers for brain development. Heatmap shows the level of expression as $\log_{10}$(FPKM+1) of literature-derived caudal vs. rostral, roof plate vs. floor plate, and midbrain-specific markers. Data from one differentiation

Disease Models & Mechanisms • Supplementary information

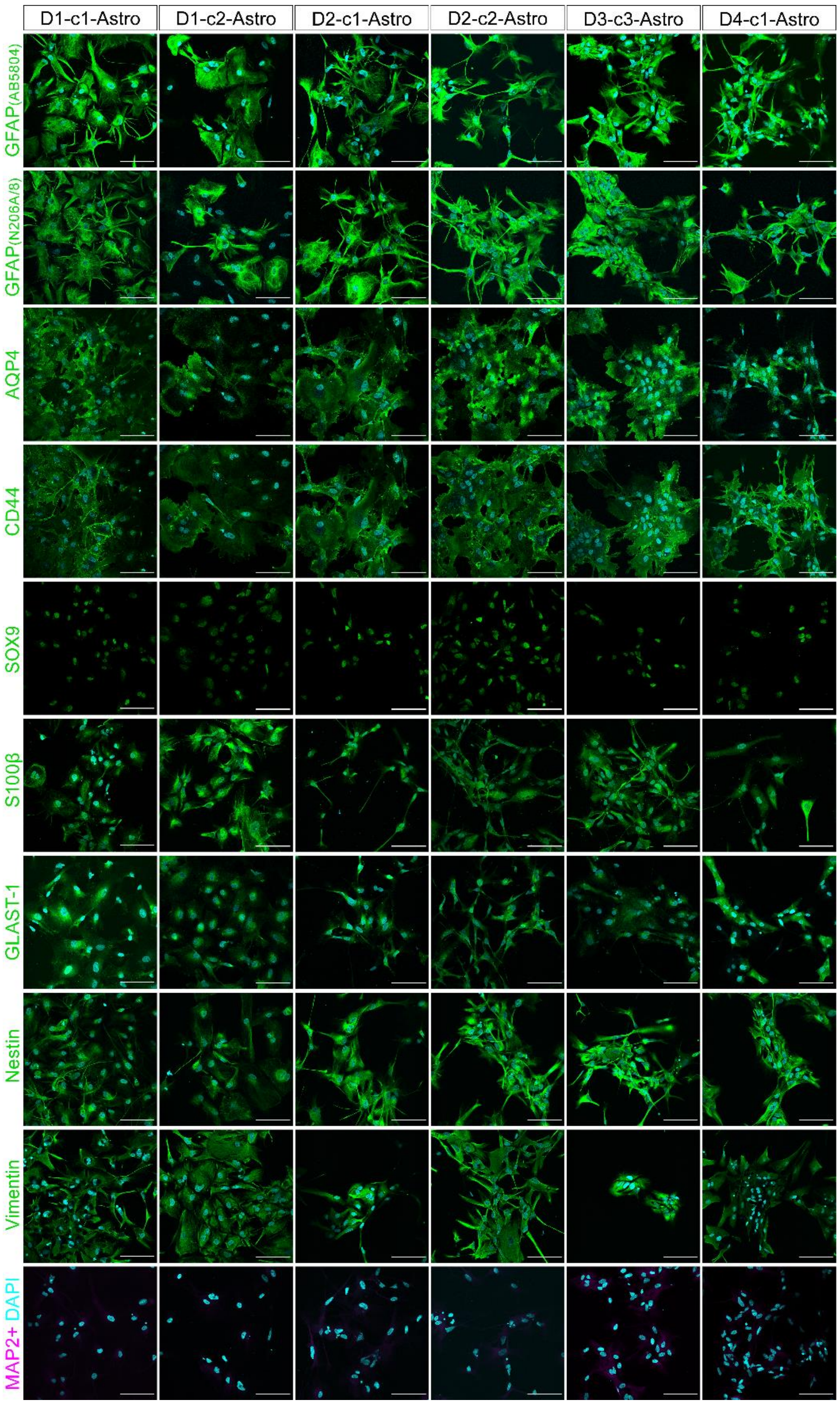

Fig. S3. **Immunocytochemical characterization of all astrocyte lines derived from ventral midbrain patterned progenitors.** Representative ICC images of 20 weeks old astrocytes staining positive for

Disease Models & Mechanisms • Supplementary information

general astrocyte markers GFAP, AQP4, SOX9, S100β, and GLAST-1, and for astrocytic precursor markers CD44, vimentin, and nestin. Stainings were negative for the mature neuron marker MAP2. Scale bars, 100 µm. Nuclei were counterstained with DAPI (cyan). Three independent differentiations for lines D1-c1, D1-c2, D2-c1, and D2-c2. Two independent differentiations for lines D3-c3 and D4-c1

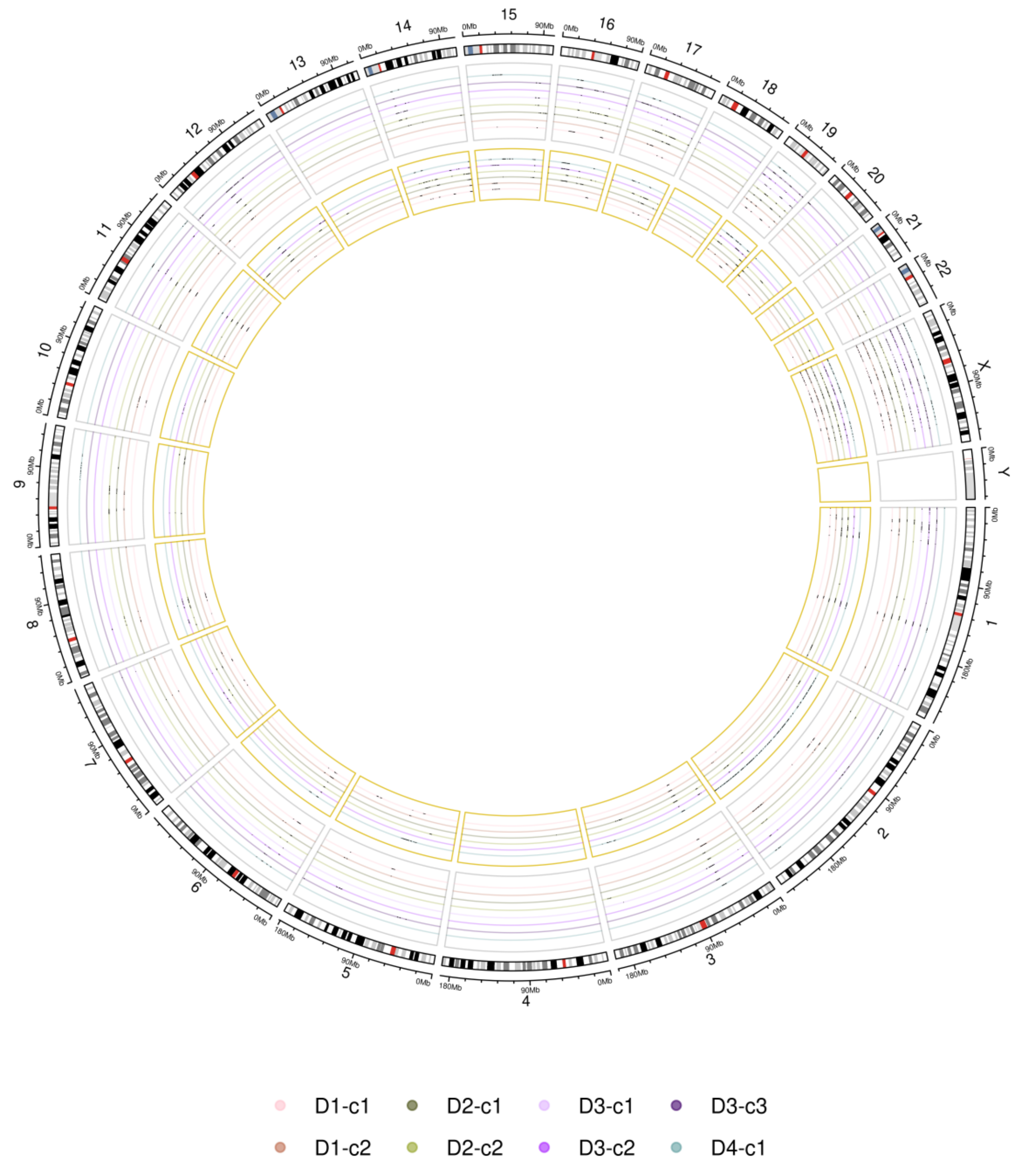

Fig. S4. **eSNP-Karyotyping reveals chromosomal multiplication in one astrocyte line.** Circle plot shows statistically significant multiplications detected by eSNP-Karyotyping (black dots). The grey inset reports the multiplications detected in the progenitors, while the golden inset those in the astrocytes

Disease Models & Mechanisms • Supplementary information

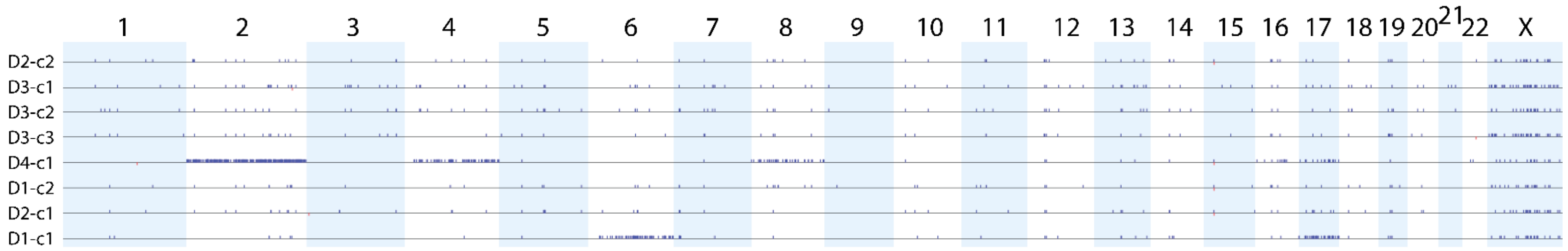

Fig. S5. **SNP-Karyotyping on genomic DNA confirms chromosomal abnormalities in the D4-c1 line and reveals abnormalities also in the D1-c1 line.** Schematic representation of the copy number variants identified in the lines. Blue lines represent copy number gains while copy number losses are reported with red lines

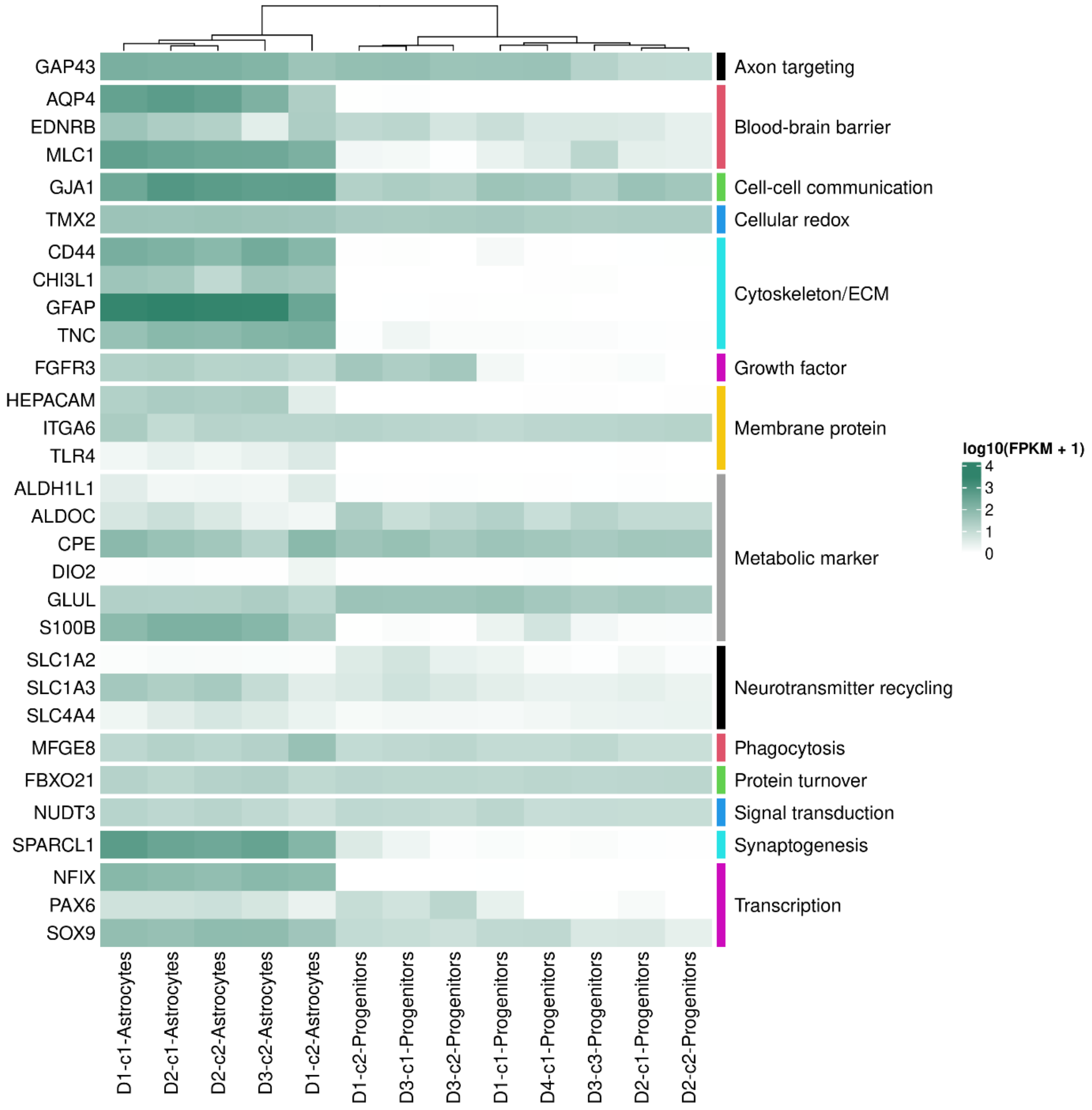

Fig. S6. **RNA-seq expression data showing enrichment of classical astrocyte markers that are known to play essential functions in astrocyte biology.** Six astrocyte lines from one differentiation were compared to eight progenitor cell lines. Heatmap shows the level of expression as $\log_{10}$(FPKM+1)

Disease Models & Mechanisms • Supplementary information

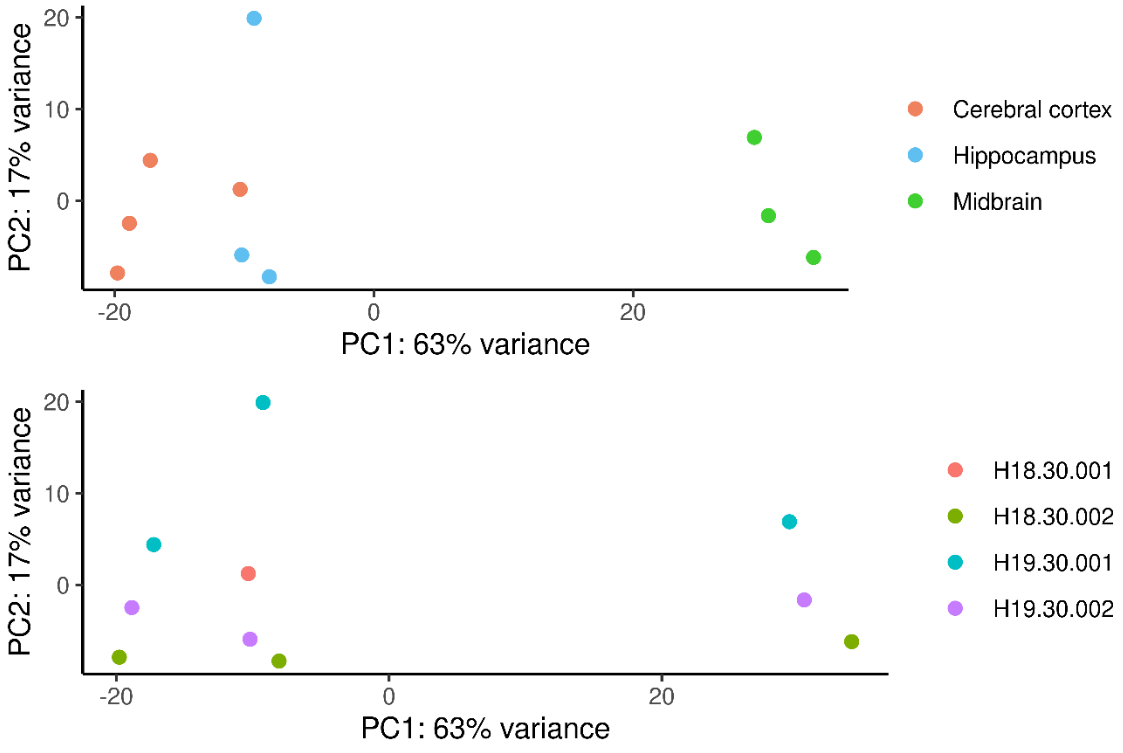

Fig. S7. **PCA plots showing clustering of pseudo-bulk region-specific samples (upper panel) or donor-specific samples (bottom panel) from recently published snRNA-sequencing (Siletti et al., 2022).** The clustering was conducted on the top 500 variable genes

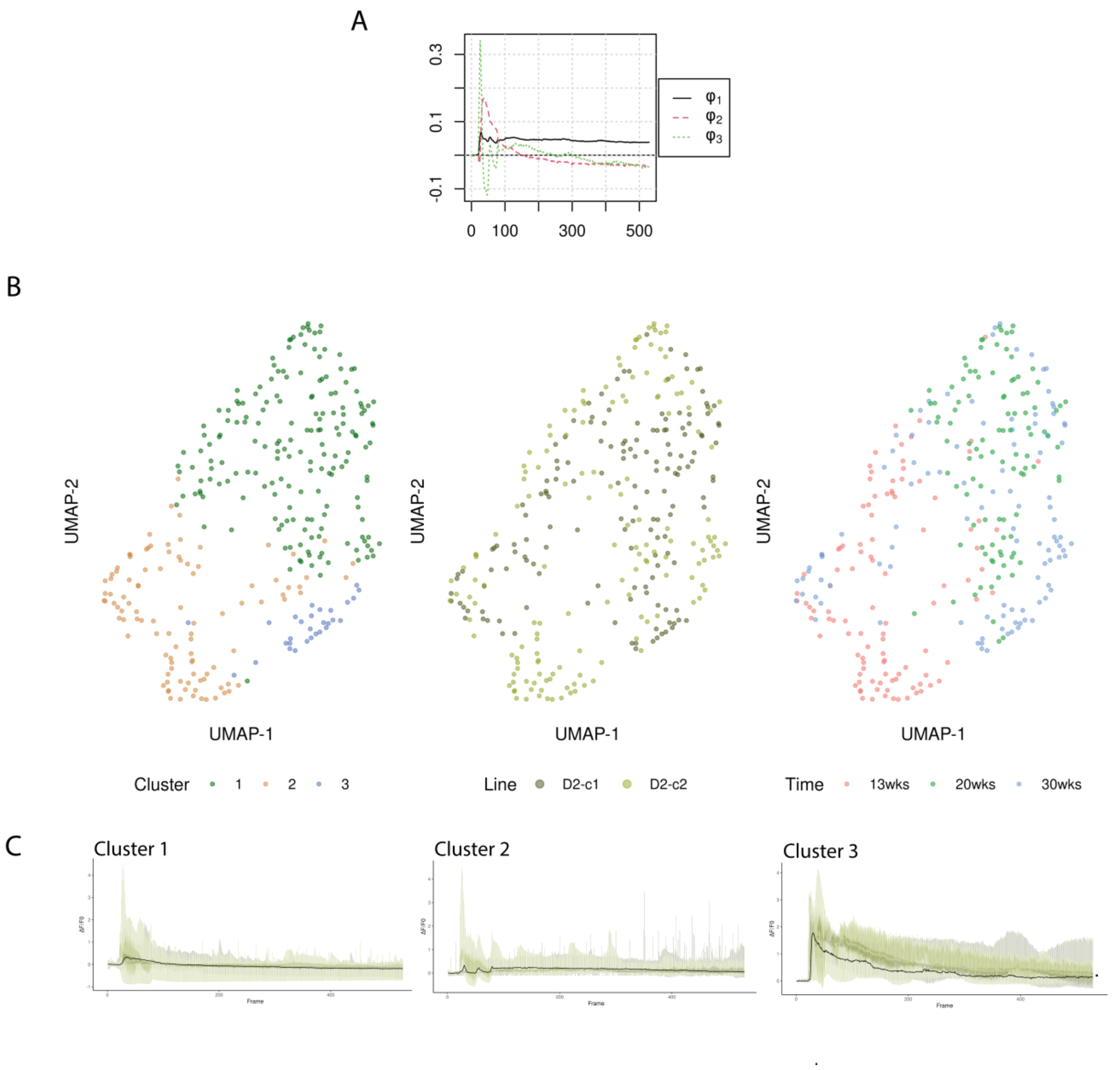

Fig. S8. **Characterization of ATP-induced Ca^{2+} transients in 13, 20, and 30 weeks old astrocytes.**

A. fPCA top 3 eigenfunctions extracted from the $\Delta F/F_0$ across the cell lines. **B.** UMAP plot showing clustering of the cells into the three k-means clusters, astrocyte lines spread, and culture time spread. **C.** $\Delta F/F_0$ profiles of the three defined clusters along the imaging time course. Tracks are colored by cell line, and the median $\Delta F/F_0$ is highlighted in black

Table S1. **Differentiation protocols to generate ventral midbrain patterned astrocytes**

Reference	Model	Progenitors	Differentiation factors	Advantages	Potential limitations
		Human iPSC-derived ventral midbrain astrocytes			
(Holmqvist et al., 2015)	2D	Dual-SMAD inhibition and patterning toward a mesencephalic fate (floor-plate progenitors) via modulation of WNT and SHH signaling as described in (Kirkeby et al., 2012)<br><br>Supplements: LDN-193189, SAG, SB-431542, SHH-C25II N-terminus, CHIR-99021	EGF, FGF-2, FBS	The high expression of astrocyte-specific markers: CD44, Connexin 43/GJA1, GLAST, NF-1A, and S100β; the bright GFAP expression can be maintained for several weeks after FACS; the secretion of pro-inflammatory cytokines and chemokines upon Il-1β and FBS treatment	The 130 days of culture; the necessity of the generation of the reporter line expressing TagRFP driven by the ABC1D element of the GFAP promoter (GFA_{ABC1D}::*TagRFP*) to obtain homogenous astrocyte populations through FACS
(Barbuti et al., 2020)	2D	Dual-SMAD inhibition and patterning toward ventral neural tube via modulation of WNT and SHH signaling, according to (Reinhardt et al., 2013)<br><br>Supplements: AA, CHIR-99021, dorsomorphin, PMA, SB-431542	CNTF, EGF, FGF-2, FGF-8, LIF, Heparin, Heregulin, VPA	Serum-free; high expression of ALDH1L1, AQP4, Connexin 43/GJA1, GFAP, S100β, and Vimentin; spontaneous cytosolic Ca^{2+} waves	The 120 days of culture; It requires many extrinsic gliogenic molecules
(de Rus Jacquet, 2019) (de Rus Jacquet et al., 2021)	2D	Dual-SMAD inhibition and patterning toward a mesencephalic fate (floor-plate progenitors) via modulation of WNT and SHH signaling as described in (Kriks et al., 2011)<br><br>Supplements: CHIR-99021, LDN193189, Purmorphamine, SB-431542, SHH-C25II N-Terminus	Commercial astrocyte medium (ScienCell)	Astrocytes are obtained in a relatively short period (~28 days)	The medium contains 2% FBS; the cells have a flat morphology; the poor immunocytochemical and functional characterization of the obtained astrocytes
(Crompton et al., 2021)	2D	Dual-SMAD inhibition and patterning toward a mesencephalic fate (floor-plate progenitors) via modulation of WNT and SHH signaling<br><br>Supplements: LDN193189, SB-431542, CHIR-99021, SHH-C25II N-Terminus	BMP4, EGF, LIF	High expression of GFAP, S100β, and ventral midbrain identity markers FOXA2, LMX1B; morphological changes and a significant increase in the secretion of IL-6 upon treatment with pro-inflammatory cytokines IL-1α or IL-1β	Time-consuming (120+ days); limited functional characterization

NF-1A, nuclear factor-1; FACS, fluorescence-activated cell sorting; RFP, red fluorescence protein; PMA, purmorphamine; AA, ascorbic acid; FGF-8, fibroblast growth factor 8; VPA, valproic acid; TGF-β, transforming growth factor beta; cAMP, adenosine-3′,5′-cyclic monophosphate; dbcAMP, dibutyryl-cAMP; SAG, smoothened agonist; NT-3, neurotrophin-3; DHA, docosahexaenoic acid

Table S2. **Cell culture media composition**

Astrocyte basal media (ABM). Keep up to one month at 4°C. Do not freeze and thaw!

DMEM-F12, GlutaMAX	100% (500 mL)	Gibco™, 10565018
N2 (100×)	1× (5 mL)	Gibco™, 17502001
B27 without vitamin A (50×)	1× (10 mL)	Gibco™, 12587010
PenStrep (100×)	1× (5 mL)	Gibco™, 15070063
MEM non-essential amino acids (NEAA) solution (100×)	1× (5 mL)	Gibco™, 11140050

Glial expansion media = ABM + supplements; added at the time of media change

HEPES	10 mM	Gibco™, 15630056
EGF	10 ng/mL	Peprotech, AF-100-15
FGF-2 (bFGF)	10 ng/mL	Peprotech, 100-18B

Glial induction media = ABM + supplements; added at the time of media change

HEPES	10 mM	Gibco™, 15630056
EGF	10 ng/mL	Peprotech, AF-100-15
LIF	10 ng/mL	Peprotech, 300-05

Glial maturation media = ABM + supplements; added at the time of media change

HEPES	10 mM	Gibco™, 15630056
CNTF	10 ng/mL	Peprotech, 450-13

Glial maintenance media = ABM + supplements; added at the time of media change

HEPES	10 mM	Gibco™, 15630056

Table S3. **Details of the primary antibodies used in this study**

**Epitope**	**Catalog**	**Source**	**Host**	**Dilution ICC**
AQP-4	HPA014784	Atlas Antibodies	Rabbit	1:200
CD44 (F10-44-2)	AB6124	Abcam	Mouse	1:200
GFAP	N206A/8	DSHB	Mouse	1:200
GFAP	AB5804	Millipore Sigma	Rabbit	1:200
GLAST-1/EAAT-1	NB100-1869	Novus Biologicals	Rabbit	1:100
NANOG	AB21624	Abcam	Rabbit	1:75
Nestin (10C2)	MAB5326	Millipore Sigma	Mouse	1:200
MAP2	188 004	Synaptic Systems	Guinea pig	1:250
OCT4	AB19857	Abcam	Rabbit	1:250
S100β	HPA015768	Sigma-Aldrich	Mouse	1:100
SOX2	AB5603	Millipore Sigma	Rabbit	1:200
SOX9	AF3075	R&D Systems	Goat	1:50
SSEA4	AB16287	Abcam	Mouse	1:75
TRA1-81	AB16289	Abcam	Mouse	1:75
Vimentin	AB92547	Abcam	Rabbit	1:200

Table S4. **Details of the 14 stem cell-derived clonal lines used in this study**

Donor ID	Source	Donor Age	Donor Sex	Donor Sampling Site	Progenitor Clone IDs	Astrocyte Clone IDs	RIN for all the clones
**D1**	Gibco™, Lot number: 1903939	33	Female	Dermal Fibroblast	c1, c2	c1, c2	10
**D2**	Gibco™, Lot number: 181388	34	Female	Dermal Fibroblast	c1, c2	c1, c2	10 (only D2-c2-Astro RIN= 8.2)
**D3**	*in house*	68	Female	Dermal Fibroblast	c1, c2, c3	c3	10
**D4**	*in house*	83	Female	Erythroid Progenitors	c1	c1	10

Table S5. **qPCR primer sequences**

Primer name	Sequence
Reactivity-specific genes	
C3-FW	AAAAGGGGCGCAACAAGTTC
C3-RV	GATGCCTTCCGGGTTCTCAA
GFAP-FW	AGAAGCTCCAGGATGAAACC
GFAP-RV	AGCGACTCAATCTTCCTCTC
LCN2-FW	ATCACCCTCTACGGGAGAACC
LCN2-RV	ACTCAGCCGTCGATACACTG
SERPINA3-FW	TGCCAGCGCACTCTTCATC
SERPINA3-RV	TGTCGTTCAGGTTATAGTCCCTC
Housekeeping genes	
CLK2-FW	TCGTTAGCACCTTAGGAGAGG
CLK2-RV	TGATCTTCAGGGCAACTCG
COPS5-FW	CCAGGAACCATTTGTAGCAG
COPS5-RV	GTAGCCCTTTGGGTATGTCC
RNF10-FW	GCATCTGTGAACTGGCTTTG
RNF10-RV	CTGACGTTTCCTCTTCTCAATG

Table S6. Results of the differential expression analysis (DEA)

Click here to download Table S6

Table S7. Results of the gene set enrichment analysis (GSEA)

Click here to download Table S7

References

Barbuti, P. A., Antony, P., Novak, G., Larsen, S. B., Berenguer-Escuder, C., Santos, B. F. R., Massart, F., Grossmann, D., Shiga, T., Ishikawa, K.-i. et al. (2020). IPSC-derived midbrain astrocytes from Parkinson’s disease patients carrying pathogenic SNCA mutations exhibit alpha-synuclein aggregation, mitochondrial fragmentation and excess calcium release. *bioRxiv*, 2020.04.27.053470.

Crompton, L. A., McComish, S. F., Stathakos, P., Cordero-Llana, O., Lane, J. D. and Caldwell, M. A. (2021). Efficient and Scalable Generation of Human Ventral Midbrain Astrocytes from Human-Induced Pluripotent Stem Cells. *J Vis Exp*.

de Rus Jacquet, A. (2019). Preparation and Co-Culture of iPSC-Derived Dopaminergic Neurons and Astrocytes. *Curr Protoc Cell Biol* **85**, e98.

de Rus Jacquet, A., Tancredi, J. L., Lemire, A. L., DeSantis, M. C., Li, W. P. and O'Shea, E. K. (2021). The LRRK2 G2019S mutation alters astrocyte-to-neuron communication via extracellular vesicles and induces neuron atrophy in a human iPSC-derived model of Parkinson's disease. *Elife* **10**.

Holmqvist, S., Brouwer, M., Djelloul, M., Diaz, A. G., Devine, M. J., Hammarberg, A., Fog, K., Kunath, T. and Roybon, L. (2015). Generation of human pluripotent stem cell reporter lines for the isolation of and reporting on astrocytes generated from ventral midbrain and ventral spinal cord neural progenitors. *Stem Cell Res* **15**, 203-20.

Kirkeby, A., Grealish, S., Wolf, D. A., Nelander, J., Wood, J., Lundblad, M., Lindvall, O. and Parmar, M. (2012). Generation of regionally specified neural progenitors and functional neurons from human embryonic stem cells under defined conditions. *Cell Rep* **1**, 703-14.

Kriks, S., Shim, J. W., Piao, J., Ganat, Y. M., Wakeman, D. R., Xie, Z., Carrillo-Reid, L., Auyeung, G., Antonacci, C., Buch, A. et al. (2011). Dopamine neurons derived from human ES cells efficiently engraft in animal models of Parkinson's disease. *Nature* **480**, 547-51.

Reinhardt, P., Glatza, M., Hemmer, K., Tsytsyura, Y., Thiel, C. S., Hoing, S., Moritz, S., Parga, J. A., Wagner, L., Bruder, J. M. et al. (2013). Derivation and expansion using only small molecules of human neural progenitors for neurodegenerative disease modeling. *PLoS One* **8**, e59252.

Siletti, K., Hodge, R., Mossi Albiach, A., Hu, L., Lee, K. W., Lönnerberg, P., Bakken, T., Ding, S.-L., Clark, M., Casper, T. et al. (2022). Transcriptomic diversity of cell types across the adult human brain. *bioRxiv*, 2022.10.12.511898.
